# Presence of Holmboe waves in particle-laden intrusive gravity current

**DOI:** 10.1038/s41598-023-34371-w

**Published:** 2023-05-02

**Authors:** Sadegh Rostami Dehjalali, Ehsan Khavasi, Parsa Nazmi

**Affiliations:** Department of Mechanical Engineering, University of Zanjan, Zanjan, Iran

**Keywords:** Mechanical engineering, Fluid dynamics

## Abstract

The present study evaluates the prevalence of Holmboe waves in an intrusive gravity current (IGC) containing particles, employing large Eddy simulation (LES). Holmboe waves, a type of stratified shear layer-generated wave, are characterised by a relatively thin density interface compared to the thickness of the shear layer. The study demonstrates the occurrence of secondary rotation, wave stretching over time, and fluid ejection at the interface between the IGC and a lower gravity current (LGC). Results indicate that, aside from *J* and *R*, the density difference between the IGC and the LGC has an impact on Holmboe instability. However, a reduction in the density difference does not manifest consistently in the frequency, growth rate, and phase speed, though it does cause an increase in the wavelength. It is important to note that small particles do not affect the Holmboe instability of the IGC, while larger particles cause the current to become unstable and vary the characteristics of Holmboe instability. Moreover, an increase in the particle diameter size results in an increment in the wavelength, growth rate, and phase speed; but is accompanied by a decrease in frequency. Additionally, the enlargement of the bed slope angle makes the IGC more unstable, encouraging the growth of Kelvin–Helmholtz waves; however, this causes Holmboe waves to disappear on inclined beds. Finally, a range for the instabilities of both Kelvin–Helmholtz and Holmboe is provided.

## Introduction

A current generated under the influence of gravity is known as a gravity current, produced by a horizontal pressure gradient that is a consequence of discrepancies in the density between two fluids or within a single fluid containing suspended particles; this type of current is referred to as a turbidity current^1^. Under certain conditions, the intrusion of a stratified ambient fluid by a density current can lead to the formation of the IGC. At the interface between the density current and the ambient fluid, instabilities such as the Kelvin–Helmholtz and Holmboe instabilities become manifest^2^. The presence of these instabilities has an influential role in determining the behavior of density currents, making them a subject of great interest in numerous scientific and engineering fields, including hydrodynamics, meteorology, oceanography, and others.

The Taylor–Goldstein equation, derived by Taylor and Goldstein utilising the theory of linear stability, was introduced to investigate the instabilities of the interface and the factors impacting it in stratified currents within the IGC. The Miles-Howard criterion, established from this equation, states that a continuous stratified current is secure if the Richardson gradient number, which is defined as the local ratio of buoyancy to inertia force, is greater than $\tfrac{1}{4}$ everywhere^3–5^. It has been demonstrated that employing Richardson’s number as the sole parameter to assess the instabilities in stratified currents is insufficient. Hazel proposed the introduction of a new parameter, denoted by *R*, to the linear stability theory, which is the ratio of the shear layer thickness $\delta _{\nu }$ to the dense layer thickness $\delta _{\rho }$^6^.

Hazel’s research presented a critical value for *R* which revealed that the Miles-Howard criterion is only valid for values lower than the critical *R*^6^. Conversely, for Richardson numbers greater than $\tfrac{1}{4}$, the Miles-Howard criterion is no longer reliable and unstable modes emerge. Holmboe was the first to theoretically uncover these modes which were later named after him, and they were found to travel in two waves with the same growth rate but opposite directions of propagation^6,7^. Smyth and Alexakis attained critically-significant *R* values of 2.4 and 2.2 respectively with the aid of numerical simulations^8,9^, while Hazel and Alexakis postulated critical *R* values of 2 through linear stability theory and analytical solutions respectively^6,10^.

Following an exploration of the conditions necessary for the generation of Holmboe waves, it has been established that they represent a classical example of shear instability resulting from the resonance between a gravitational wave and a vorticity wave, which is present in both symmetric and asymmetric cases^11^. When the velocity and concentration profiles are concentric, meaning that their inflection points are the same, two unstable modes with the same growth rate and phase speed but moving in opposite directions, known as symmetric Holmboe case, are created^12^. The velocity and concentration profiles being non-concentric results in the growth rates of the two unstable Holmboe modes being altered variably, leading to one of them having a higher growth rate and thus dominating the current. This type of asymmetric instability is referred to as One-Sidedness^13^. Subsequently, Haigh and Lawrence discovered that the most unstable mode is manifested in higher wave numbers when the asymmetry increases, an effect which was later corroborated by Carpenter et al., who additionally demonstrated an augmentation in mixing rates^14,15^.

Holmboe Waves are significantly less frequently encountered than Kelvin–Helmholtz Waves, owing to the numerous restrictions on their generation; nevertheless, they remain a powerful force in the realm of geophysical currents and have been observed in the natural world^8^. In comparison to Kelvin–Helmholtz waves which remain static, these waves possess a non-zero phase speed and, similarly to Kelvin–Helmholtz waves, the formation and dissipation of these waves exhibit secondary rotations or instabilities^15^. The Holmboe instability was investigated using a combination of analytical, experimental, and linear stability analysis techniques, as elucidated in the following paragraphs.

Numerous laboratory studies have been conducted to investigate Holmboe waves, the outcomes of which are documented herein. A substantial amount of laboratory research has been conducted to observe the existence of Holmboe waves; however, most experiments only yielded very faint indications of symmetric Holmboe waves (e.g. Lefauve et al. and Zhu and Lawrence), likely due to the technical constraints of the experimental process^13,16^. Asymmetry in the velocity and concentration profiles observed in laboratory settings contributes to the emergence of One-Sidedness as reported in^17^. This phenomenon places certain constraints upon the conduct of laboratory studies. Tedford et al. conducted a meticulous analysis of Holmboe waves in a rectangular cross-sectional channel of extended length, assessing parameters such as phase speed, wave number and wavelength^12^. Comparisons were then made between the observed results and those predicted analytically. Yang et al. conducted a recent experimental study, in which the formation of asymmetric Holmboe instabilities on an arrested salt wedge was revealed^18^. Subsequently, linear stability theory was used to deduce the growth of the said instabilities^18^. The findings of the study revealed that the Holmboe wavelength increases downstream along the salt wedge^18^. Such wave stretching is attributed to the increase in the shear layer thickness, which is further reinforced by the gradual acceleration of the upper layer fluid^18^.

The comparative study of instabilities has predominantly been conducted using theoretical or numerical simulations, as these methods enable the analysis of parametric effects on instability. Carpenter et al. directly compared laboratory and numerical methods through direct numerical simulation (DNS) of the said channel in the work of Tedford et al.^12,19^. According to Salehipour et al., the turbulent characteristics and structures of a flow with Holmboe waves at high Reynolds numbers differ significantly from those of a flow with Kelvin–Helmholtz waves, as established through the DNS^20^. By means of utilizing the direct two-dimensional simulation method in combination with two miscible fluids (heavy fluid below), Zagvozkin et al. discovered the presence of Holmboe and Kelvin–Helmholtz waves, consequently implying the instability of the interface between the miscible currents^21^. Additionally, Parker et al. employed the linear stability theory to study the effect of various parameters, such as viscosity, on the Holmboe waves, and discovered that even in low values of *R*, viscosity-induced Holmboe waves (termed as viscous Holmboe waves) arise^11^.

In the ongoing analysis of Holmboe waves, the Boussinesq approximation can be referred to in order to gain insight. Churilov, without relying on the Boussinesq approximation, examined the Holmboe instability in a sharply stratified shear flow^22^. He devoted special attention to the disarticulation, as well as the analysis of the role of instability resulting from wave-wave and wave-particle interactions, which are the two fundamental physical mechanisms responsible for the loss of stability. He established that both of these mechanisms are of equal importance for a better understanding of the instability’s features^22^. In order to gain insight into the inertial effects of variations in density, Guha and Raj examined a range of non-Boussinesq shear flows and Holmboe waves, which are a type of steadily propagating wave that can occur at the boundary between two fluids with different background densities and vorticities^23^. Some of the results, such as the destabilizing nature of density stratification and the stabilizing effects of shear, appeared to be counterintuitive; however, these instabilities could be explained in terms of wave-interaction if viewed from a different perspective^23^.

The Reynolds Averaged Navier-Stokes (RANS) model is not suitable for representing the intricate details of surface phenomena, and therefore does not serve as an appropriate choice for numerical simulations of interface interactions. Although Direct Numerical Simulations are highly accurate and show all the details, they are limited by the requirement for sophisticated hardware and have an exceedingly long computational time and cost^1^. Since the majority of numerical investigations have been performed using DNS, this study opts for the use of the Large Eddy Simulation (LES) method, which is adequate to display and analyze the details of the flow and has a comparatively shorter computation time and cost than DNS.

The present study is the first to investigate the Holmboe waves in an IGC containing particles. This is particularly significant as numerous researchers have already studied the instability of Holmboe in stratified shear flows, yet this phenomenon has yet to be examined in the case of the IGC. In addition, the various aspects of this instability have been extensively considered and tested. Rostami DehJalali et al. briefly explored this topic in the context of the particle-free (PF) IGC and the results of this are compared to those from the particle-laden (PL) IGC in the current study^24^. To this end, Large Eddy Simulation (LES) is employed to simulate both the particle-free and particle-laden IGCs within the channel.

The primary aim of this research is to gain a deeper understanding into the instability of Holmboe and to investigate the influence of a range of parameters on it in the IGC. To ascertain the accuracy of the findings, they are compared with those reported by Khodkar et al. which were obtained by means of a DNS^2^. In “The model” section governing equation and modeling approaches are presented. Validation of the simulation is demonstrated in “Model verification” section. “Results and discussions” section is focused on discussion and comparison of the numerical results and finally, conclusions of the Holmboe investigation in IGC are expressed in “Conclusion” section. At the end of the manuscript, a compilation of acronyms used throughout the article is presented in “Abbreviations” section to facilitate and enhance the reading experience.

## The model

**Figure 1:**
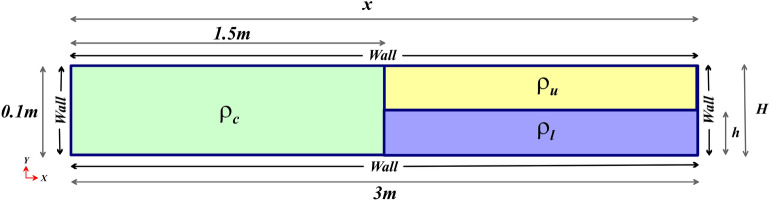
A two-dimensional schematic of the simulated channel geometry is presented. An intrusive fluid of density $\rho _{c}$ (IGC) is situated to the left of the channel, while two ambient fluids of densities $\rho _{u}$ (UGC) and $\rho _{l}$ (LGC) are placed to the right of the channel, with a plane at a distance of 1.5 m from the left side wall serving as a separator.

This study is performed in a two-dimensional channel of length 3 m and height 0.1 m (Fig. 1). The upper and lower boundaries are assumed to be wall types, and due to the lack of inlets and outlets, the left and right boundaries are also considered wall types. An intrusive fluid of density $\rho _{c}$ is located to the left of the channel, while two ambient fluids of densities $\rho _{u}$ and $\rho _{l}$ are located to the right of the channel, separated by a plane at a distance of 1.5 m from the left side wall.

These non-dimensional numbers are utilized in order to facilitate a more thorough comprehension and visualization of the outcomes:

1 $$\begin{aligned}&x^{*} = \frac{x}{x_{ref}} \end{aligned}$$

2 $$\begin{aligned}&t^{*} = \frac{t}{t_{ref}} \end{aligned}$$

3 $$\begin{aligned}&U^{*} = \frac{U}{U_{ref}} \end{aligned}$$

The above equations $x^{*}$, $t^{*}$, and $U^{*}$ denote dimensionless position, time, and velocity respectively, while $x_{ref}$, $t_{ref}$, and $U_{ref}$ are defined as *H*, $H/\sqrt{g^{'} \cdot H}$, and $\sqrt{g^{'} \cdot H}$ respectively. Consequently, the computational domain is characterized by a dimensionless length of 30, a height of 1, and the plane location of 15. All numerical simulations are conducted with a Reynolds number greater than 1000, thereby eliminating the effect of viscosity and ensuring that the current is turbulent^25^. In this setup, the Boussinesq approximation is assumed, along with the Newtonian properties of the fluid, as well as the two-dimensional current and incompressible hypotheses.

By disregarding the *z*-coordinate in the governing equations, it can be ascertained that Holmboe waves are two-dimensional since they move along the length of the channel, increasing or decreasing in height. This two-dimensional nature of the Holmboe instability was theoretically demonstrated by Haigh and Lawrence and experimentally verified by Khavasi and Firoozabadi^14,26^. However, Smyth postulated that at very low Reynolds numbers, ranging from 200 to 400, three-dimensional instability is observed^27^. This was further corroborated by Carpenter et al. (2010) through a three-dimensional numerical simulation which illustrated that even at Reynolds numbers of about 600, Holmboe waves remain two-dimensional^19^.

The current investigation utilizes the LES approach wherein a spatial filter is applied to the equations, in this case a box filter, to compute the effects of large eddies on the flow while directly modelling the impact of the small eddies. The final governing equations of the PL IGC in its filtered form comprise the continuity, momentum, and concentration equations as detailed in^28^:

4 $$\begin{aligned}&\dfrac{\partial u_j}{\partial x_j} = 0 \end{aligned}$$

5 $$\begin{aligned}&\dfrac{\partial u_i}{\partial t}+u_j\dfrac{\partial u_i}{\partial x_j} = \nu _w\dfrac{\partial ^2 u_i}{\partial x_j\partial x_j} + \dfrac{\partial }{\partial x_j}(2\nu _{SGS} S_{ij}) - \dfrac{1}{\rho _w}\dfrac{\partial p}{\partial x_i} - g^{'}(c_1+c_2)\delta _{2i} \end{aligned}$$

6 $$\begin{aligned}&\dfrac{\partial c_1}{\partial t} + u_j\dfrac{\partial c_1}{\partial x_j} = \xi \dfrac{\partial ^2 c_1}{\partial x_j\partial x_j} + \dfrac{\partial }{\partial x_j}\left( \xi _{SGS}\dfrac{\partial c_1}{\partial x_j}\right) + V_s\dfrac{\partial c_1}{\partial x_j}\delta _{2i} \end{aligned}$$

7 $$\begin{aligned}&\dfrac{\partial c_2}{\partial t} + u_j\dfrac{\partial c_2}{\partial x_j} = \xi \dfrac{\partial ^2 c_2}{\partial x_j\partial x_j} + \dfrac{\partial }{\partial x_j}\left( \xi _{SGS}\dfrac{\partial c_2}{\partial x_j}\right) \end{aligned}$$

The equations presented herein feature two constants, $\rho _{w}$ and $\nu _{w}$, representing the density and kinematic viscosity of the ambient fluid (water), respectively. Moreover, the variables $c_{1}$, $c_{2}$, $\xi$, and $\xi _{SGS}$ denote the concentration of the density current, concentration of ambient fluid, molecular diffusivity, and the Sub-Grid Scale (SGS) molecular diffusivity, respectively. Additionally, $S_{ij}$ is the strain rate tensor and $\delta _{2i}$ is the Kronecker delta; it should be noted that the direction of gravity is opposite to that of the *y* or $i = 2$ direction.

The motion of gravity currents is primarily determined by the contrast in density between the two fluids. This can be described by the initial reduced gravity acceleration $g^{'}$, which is defined as follows:

8 $$\begin{aligned}&g^{'} = g\beta \end{aligned}$$

9 $$\begin{aligned}&\beta = (\rho _{max} - \rho _w)/ \rho _w \end{aligned}$$

10 $$\begin{aligned}&c = (\rho - \rho _w)/(\rho _{max} - \rho _w) \end{aligned}$$

In Eq. (9), a distinction is made between the maximum density, $\rho _{max}$, and the mixture density, $\rho$. The Boussinesq approximation was utilized in Eqs. (4)–(7), as the difference between the densities of the light and heavy fluids (water and salt water, respectively) in the simulations of this research was slight ($2.1\%$). This approximation is commonly employed in calculations of this nature, as evidenced by the research conducted by Ooi et al.^29^; In line with this assumption, the effects of density variations on the momentum equation are only applicable in the gravity term.

In Eq. (5), a closure is required to model the modified residual stress tensor or modified SGS tensor through the use of the LES. The Smagorinsky model is the simplest model and serves as the basis for more advanced models. In this study, the Wall-Adapting Local Eddy-Viscosity (WALE) model is employed, which is a newer model compared to the Smagorinsky and Smagorinsky dynamic models for multiple simulations in complex geometries. The Smagorinsky and Smagorinsky dynamic models only calculate the SGS stress based on the strain rate tensor. However, this definition is not accurate when dealing with significant rotation rates, such as secondary rotations due to Holmboe waves^17^. The WALE model, which is based on the traceless symmetric part of the square of the velocity gradient tensor, takes both rotation and strain effects on turbulence structures into account^30^.

The Schmidt number, $Sc = \nu /\xi$, is the ratio of the kinematic viscosity to the molecular diffusivity and its value is considered to be $Sc = 1$ when $Sc \ge 1$, as is often the case in the concentration equations (Eqs. 6 and 7)^28,29^. Similarly, the turbulent Schmidt number ($Sc_{SGS}$) is the ratio of turbulent (Eddy) viscosity ($\nu _{SGS}$) and turbulent (Eddy) molecular diffusivity ($\xi _{SGS}$). Despite 1 being often used as a reasonable approximation for $Sc_{SGS}$ in engineering contexts, such a value should be taken with caution as it varies according to spatial and temporal fluctuations and is dependent on the parameters of the problem to be addressed, which can even take on negative values locally^31^.

Donzis et al. conducted an extensive analysis of a substantial database generated from DNS of passive scalars sustained by a homogeneous mean gradient and mixed by homogeneous and isotropic turbulence, and extracted the values of $Sc_{SGS}$ over an expansive range of the Taylor micro-scale Reynolds number $Re_{\lambda }$ (formed with the root-mean square turbulent velocity and the Taylor micro-scale) of 8–650, and *Sc* of 1/2048 to 1024. This range of Schmidt number encompasses mostly environmental contaminants. It was proposed that the turbulent Schmidt number is a function of $Re_{\lambda }$ and *Sc*, and the results of the analysis revealed that $Sc_{SGS}$ has a unique functional dependence with respect to the molecular Péclet number (*Pe* = $Re_{\lambda }^2$ * *Sc*). Furthermore, for $Pe < 100$, $Sc_{SGS}$ was larger than 2 and asymptotically increased with the decreasing *Pe*, and for $Pe > 1000$, $Sc_{SGS}$ attained a constant value of approximately 1–1.3^31,32^. The Péclet number, which was ascertained to surpass 1000 in the present research, supports employing a fixed value of unity for the turbulence Schmidt number. Finally, sub-grid scale Eddy viscosity, $\nu _{SGS}$, can be modeled through the LES and based on the turbulent Schmidt number, $Sc_{SGS}=1$^31–34^, the turbulent molecular diffusivity, $\xi _{SGS}$, can be derived using the following expression^28^:

11 $$\begin{aligned} \xi _{SGS} = \dfrac{\nu _{SGS}}{Sc_{SGS}} \end{aligned}$$

The IGC employs Eq. (6) as its concentration equation, which incorporates a settling velocity ($V_s$). In contrast, the stratified ambient current concentration equation (Eq. 7) does not take into account settling velocity as particles are assumed to be a fluid with vertical settling velocity. To approximate the settling velocity in low Reynolds numbers, the Stokes drag force is employed. Among the equations used for calculating the Stokes settling velocity, Eq. (12) is one of the most crucial^35^. By applying this equation to the concentration equation (Eq. 6), the effect of the particles on the concentration is accounted for.

12 $$\begin{aligned} V_s = gd^2_P\dfrac{\rho _p - \rho }{18\mu } \end{aligned}$$

In the equation above, $\rho _p$ and $d_p$ represent the density and diameter of particles, respectively. For the purpose of PL simulations, it is assumed that all particles are comprised of Kaolin with a density of $\rho _p = 2650$ kg/m$^3$, and interactions between particles are disregarded.

The behavior of the seeding material utilized in the simulations can be assessed by calculating the particle Stokes number ($St_d$), which expresses the ratio of the response time of the particle ($\tau _p$ = (1/18)($\rho _p/\rho _w$)($d_p^2/\nu$)) to the turbulent dynamic time at the scale of the particle size ($\tau _d$ = ($d_p^2/\epsilon$)$^{1/3}$)^36,37^. Specifically, Stokes numbers higher than 1 signify that the particles detach from the flow, while for Stokes numbers lower than 1 the particles remain closely associated with the fluid streamlines. Consequently, the Stokes number can be formulated as follows^37^:

13 $$\begin{aligned} St_d = \frac{\tau _p}{\tau _d} = (1/18)(\rho _p/\rho _w)(d_p^2/\eta ^{4/3}) \end{aligned}$$

The Kolmogorov length-scale, denoted by $\eta$, is equal to ($\nu ^3/\epsilon$)$^{1/3}$, with $\epsilon$ being the turbulent dissipation rate. The resultant Stokes numbers, as calculated herein, are presented in Table 1. These outcomes demonstrate that, for the particular cases examined, the particles are closely adhering to the flow.

**Table 1:** Evaluated Stokes numbers for the PL assumptions.

$d_p$	12 $\upmu$m	20$\upmu$m	30 $\mu$m
$St_d$	$1.41 \times 10^{-5}$	$3.91 \times 10^{-5}$	$8.81 \times 10^{-5}$

The discretization of divergence terms in the governing equations is achieved by utilizing the methods of Gauss linear, Gauss QUICK, and Gauss limited linear, while the discretization of Laplacein terms is addressed by the modified Gauss linear. The gradient term is tackled with the method proposed by Peer et al., and a backward second order method is employed for time discretization^38,39^.

## Model verification

In order to validate the solver utilized, a geometry analogous to the studied example of Khodkar et al. is constructed^2^. Since the height of the ambient fluid influences the symmetry or asymmetry of the current, the validation of each is investigated in two independent sections and the attained outcomes are contrasted and examined (see “Validation”) with the DNS results of Khodkar et al.^2^. To acquire the number of computational nodes and the length of each cell in different directions, the research conducted by Pelmard et al. on mesh quality was consulted^40^. According to this investigation, in LES, the greatest mesh size scale is obtained with *H*/16 whereby *H* is the height of the current, and the minutest, which is required to be greater than the Kolmogorov length scale, is realized with $(H/2){Re}^{-3/4}$^40^. Thus, taking into account the values of *H* and *Re*, information of the mesh employed in the present study is provided in Table 2. To guarantee the quality of the mesh, diagrams of $Y^{+}$ and power spectra are produced for a critical simulation (slope of 6 degrees).

**Table 2:** Details of division and cells number which are considered for mesh.

	Streamwise	Bottom wall-normal	Spanwise	Total number of cells
Computational grid	1350	270	1	364,500

### $Y^{+}$ diagram

The modeling of velocity profiles on solid boundaries is of great significance since it affects the shape of the flow patterns and the forces acting on them. Nevertheless, it is impossible to model the numerous small eddies that occur near the solid boundaries due to turbulent flow. The boundary layer comprises three distinct layers: the innermost one is the viscous sublayer, where the flow is laminar and molecular viscosity is the primary factor in momentum equations; the outermost is a fully turbulent region, where turbulent shear stress is the main contributor; and the middle layer, or buffer layer, is the transition area between laminar and turbulent, where both turbulent and viscous shear stresses are significant^41^.

The height of the initial cell adjacent to the solid boundary can be reduced in order to incorporate the influence of molecular viscosity into the computations. According to Eq. (14), $Y^+$ is the dimensionless height of this first cell, and the friction velocity can be determined through the application of Eq. (15)^40^.

14 $$\begin{aligned} Y^+= & {} \dfrac{\Delta y U_{fric}}{\nu } \end{aligned}$$

15 $$\begin{aligned} U_{fric}= & {} \sqrt{\dfrac{\tau _ w}{\rho }} \end{aligned}$$

**Figure 2:**
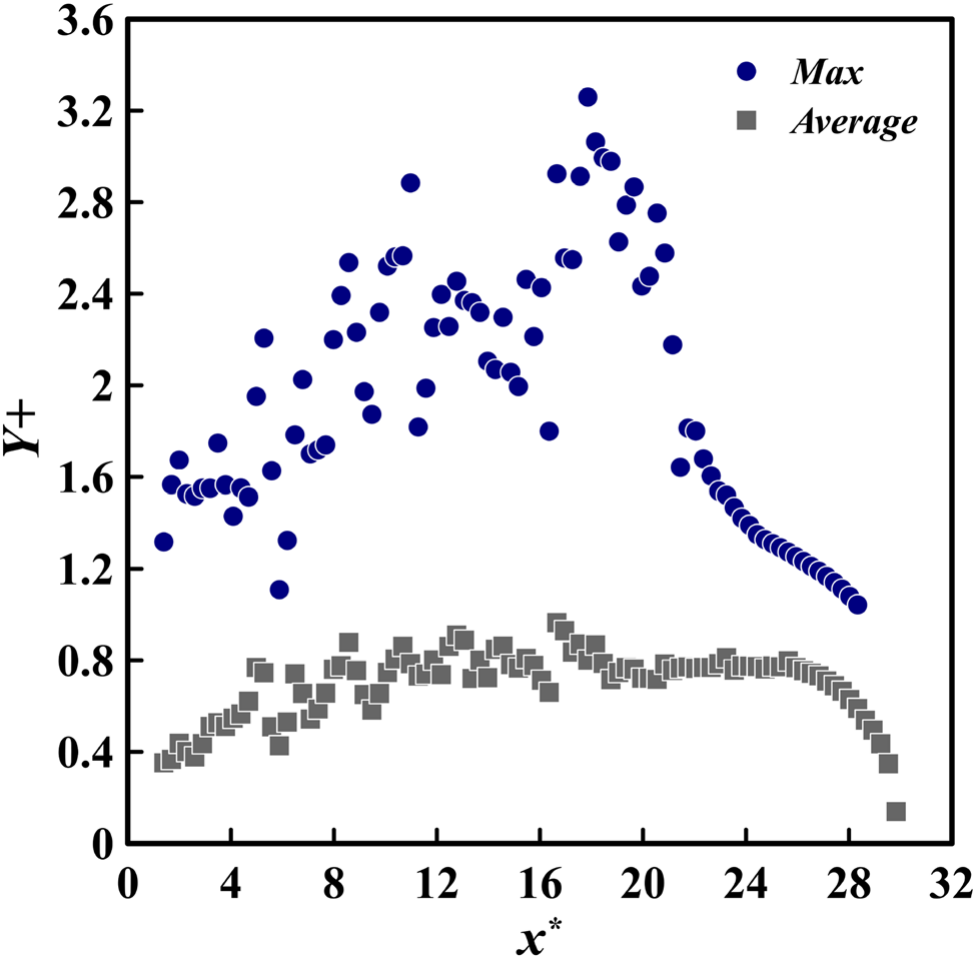
The maximum and average values of $Y^+$ for the critical simulation computational grid (slope of $6^{\circ }$) are observed to be less than 10 and 1 respectively. The $c_1$ and $h^*$ characteristics of the critical state are determined to be 0.3, when the PF assumption is taken into consideration.

The results of the present current simulation containing LES reveal that the maximum values of $Y^+$ in the viscous sublayer should be no more than 10^40^. This is illustrated in Fig. 2, which shows the maximum and mean $Y^+$ values for the mesh specified in Table 2 for a critical simulation (slope of 6 degrees). Evidently, the maximum value of $Y^+$ is less than 10 and the average is below 1, indicating that the mesh is appropriate for the simulation and able to accurately capture the viscous sublayer.

### PSD diagram

The wide range of turbulence scales, from the largest eddies that interact with the mainstream to the smallest eddies where energy dissipation occurs, is indicative of the energy cascading relationship between them. Moreover, in accordance with the Kolmogorov hypothesis, it is assumed that, at high Reynolds numbers, the power spectra density (PSD) has a range of wave numbers between the wave numbers of large eddies and the Kolmogorov wave numbers (small eddies). This range is identified by the dissipation rate, and is not affected by the molecular viscosity, dubbed the inertial subrange^41^.

**Figure 3:**
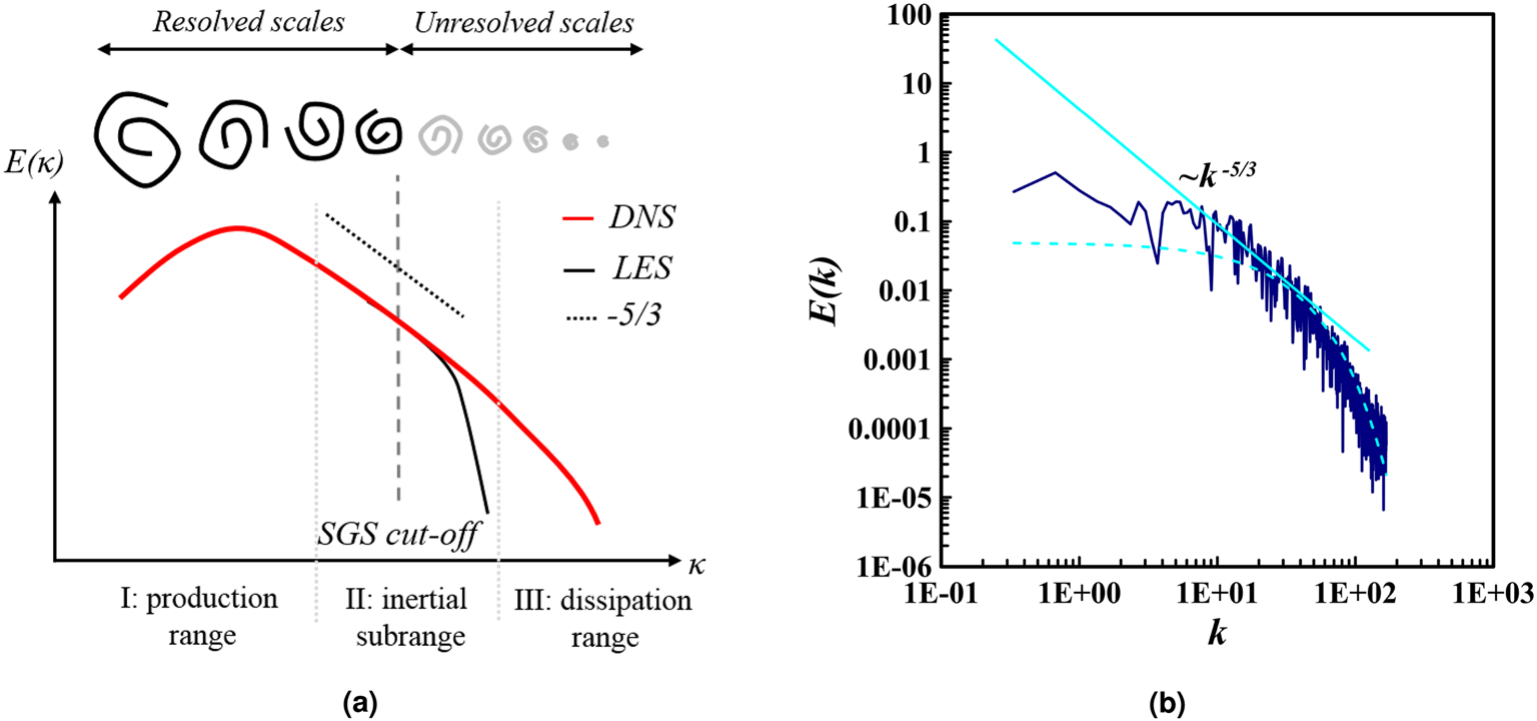
Figure (**a**) reveals an example of the eddies energy spectrum^42^. The power spectra of the eddies for the current investigation’s critical simulation on a computational grid with a 6-degree slope is illustrated in (**b**). The presence of a slope of $-5/3$ in the PSD diagram of the critical simulation (**b**) serves as an affirmation of the mesh’s adequacy. The $c_1$ and $h^*$ characteristics of the critical state are determined to be 0.3, when the PF assumption is taken into consideration.

The PSD diagram (Fig. 3a) can be used to ascertain the energies of eddies for various frequencies and wavelengths. The presence of an inertial subrange in the solution domain is a sign of a turbulent flow, and the mesh quality can be considered satisfactory when the energies of small and large eddies are met. Additionally, the difference between the LES and DNS methods can be observed in Fig. 3a. In the current study, a slope of $-5/3$ is visible in the PSD diagram of the critical simulation (Fig. 3b) which serves as confirmation of the mesh quality, as presented in Table 2. This further corroborates the turbulent nature of the flow.

### Validation

**Figure 4:**
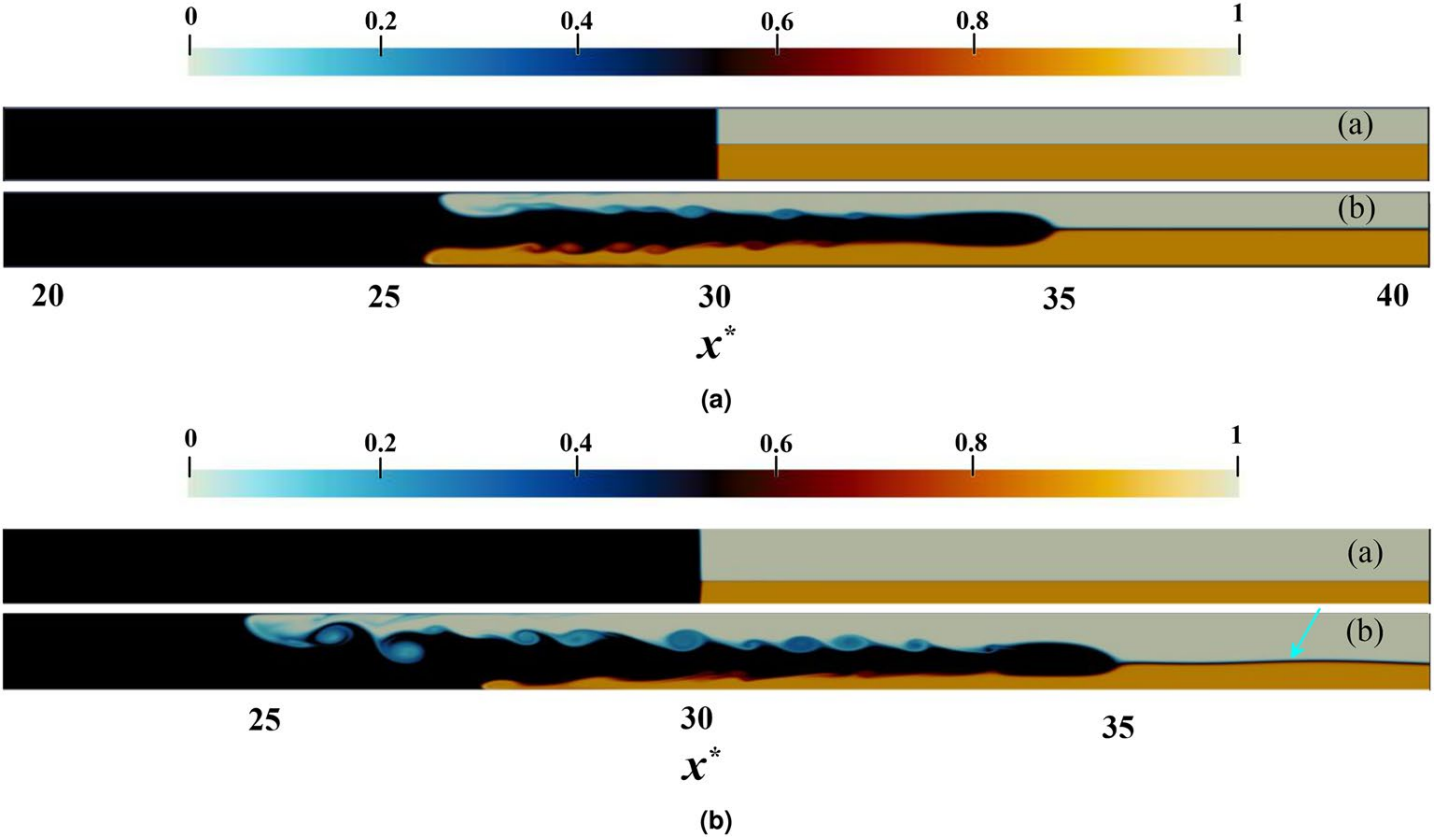
The graphical representations depicted in (**a**) and (**b**) illustrate that for $c_1 = 0.5$, a doubly symmetric IGC motion is observed when $h^{*} = 0.5;$ conversely, an asymmetric IGC motion is perceived when $h^{*} = 0.3$ for dimensionless times (**a**) 0 and (**b**) 20. The variation in color is indicative of an alteration in the relative density of IGC, UGC and LGC, as represented by the color band. The density field varies from 0 to 1.

After conducting an assessment of the computational grid quality, the present study perform validation of the solver for two simulations with both a doubly symmetrical and asymmetrical assumption. The former involve an IGC density equal to the average density of the ambient fluids, as well as heights of the ambient fluids being identical. Figure 4a in the current assessment demonstrates the motion of the IGC in the simulation where $c_1 = 0.5$ and $h^{*} = 0.5$ for both 0 and 20 dimensionless times. In this instance, the velocities of the LGC and upper gravity current (UGC) are equal and the interface of the IGC and ambient fluids is observed to be both smooth and uniform.

**Figure 5:**
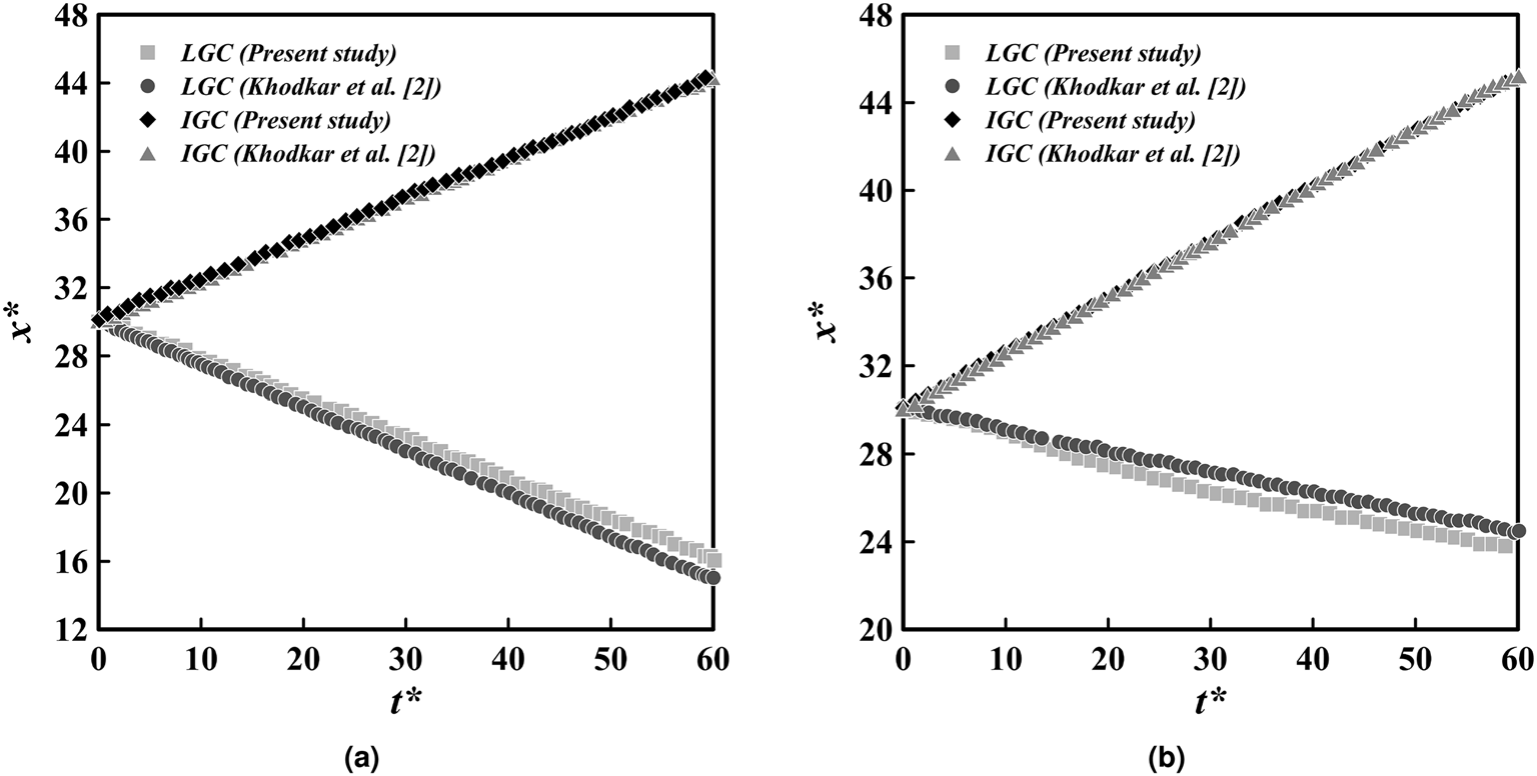
A comparison between the IGC and the LGC head position diagrams, with regards to both the doubly symmetric (**a**) and the asymmetric (**b**) assumptions, with the research conducted by Khodkar et al.^2^ is performed. The $c_1$ and $c_2$ thresholds for calculating the head position are set to 0.0001.

The head positions of the IGC and LGC are extracted under the assumption of double symmetry, which is then compared with the results of Khodkar et al.^2^. This comparison is illustrated in the Fig. 5a. The mean and maximum relative discrepancies between the present study and the results of Khodkar et al. for the IGC are estimated to be $0.5\%$ and $0.8\%$, respectively, and for the LGC are measured to be $3.5\%$ and $5.0\%$, respectively, which indicates a satisfactory level of agreement^2^.

The asymmetric assumption in the simulation results in the IGC density being the average density of the ambient fluids, however the height of the ambient fluids differ. In the simulation conducted for the present inquiry, with $c_1 = 0.5$ and $h^{*} = 0.3$, the motion of the IGC is depicted in Fig. 4b at 0 and 20 dimensionless times. In this case, the UGC and LGC head positions along the upper and lower walls show variation. Additionally, the interface of the two ambient fluids exhibits a wave formed by a progressive IGC, as indicated by the blue arrow in Fig. 4b, which moves at a higher speed than the IGC itself.

The comparison of the head position of the IGC and the LGC under the asymmetric assumption, as depicted in Fig. 5b, is in agreement with the results of Khodkar et al. with a mean relative difference of $0.4\%$ for the IGC and $4\%$ for the LGC, and the maximum value of $0.56\%$ and $6.5\%$, respectively^2^. The accuracy of the simulated results can be assured by considering both doubly symmetric and asymmetric cases.

## Results and discussions

The results of the study are divided into five key components, which are discussed in the “wave evolution” subsection with respect to the formation and features of Holmboe waves in the IGC under the PL case. Subsequently, in the following three subsections, the effects of “the IGC density”, “particle size” and “inclined bed” on the various characteristics of Holmboe waves (R and J, frequency, growth rate, phase speed and wavelength) are examined. Ultimately, in the last subsection, based on the results and simulations conducted in the earlier subsections, the R-J diagram is used to determine an instability boundary between Holmboe and Kelvin–Helmholtz waves in the IGC, which is referred to as “the transition range in the IGC”.

### Wave evolution

The present investigation is revealed the occurrence of the one-sidedness phenomenon due to the asymmetry of the velocity and concentration profiles, rather than the symmetric Holmboe waves. The vorticity in both layers is generated by the presence of the shear force on the interface, and for intensive stratified media (i.e. large Richardson numbers) rotations are produced in both layers due to the shear stress, which are prevented from joining and forming a vorticity train by the large density difference. Subsequently, the Holmboe waves are formed in each layer by the movement of these rotations, which appear and disappear at different time intervals, and occasionally two or three waves may be combined and a large volume of the LGC fluid is forced into the IGC.

**Figure 6:**
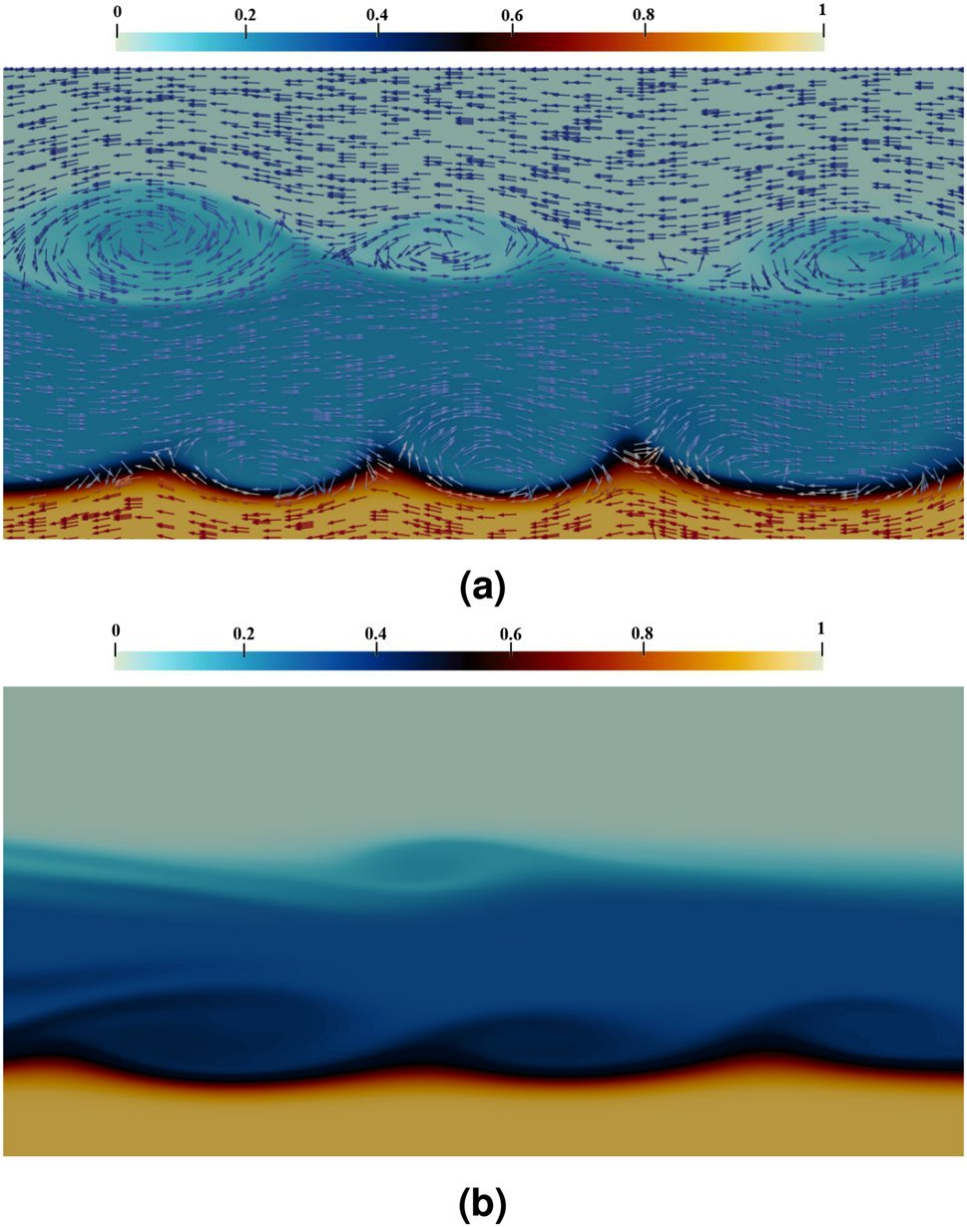
The velocity vectors and interface between the IGC and the reverse currents for a simulation wherein $c_{1} = 0.2$, $h^{*} = 0.3$ and $d_{p} = 12\,\upmu$m at $t^{*} = 42$ can be seen in (**a**). Additionally, (**b**) illustrates the presence of secondary rotations when $c_{1} = 0.3$, $h^{*} = 0.3$ and $d_{p} = 12\,\upmu$m at $t^{*} = 42$ is considered in the simulation. The range of colors displayed is suggestive of a disparity in the proportion of IGC, UGC, and LGC, as symbolized by the color band. This density field has a range of values between 0 and 1.

The position of the interface between the IGC and the reverse current, as well as the velocity vector, can be seen in Fig. 6a for a simulation with parameters $c_{1} = 0.2$, $h^{*} = 0.3$, and $d_{p} = 12\,\upmu$m. Holmboe waves are observed at the interface between the IGC and the LGC, generating a region of high vorticity which moves slower than the IGC, creating a high vorticity region in the lee of the wave crest. Additionally, Kelvin–Helmholtz waves take shape at the interface between the IGC and the UGC, with a smaller influence region than Holmboe waves. It is worth noting that the streamlines become closed in the region of high vorticity when the Kelvin–Helmholtz wave is generated, in addition to the secondary rotations observed in the present investigation with Holmboe waves (Fig. 6b), as documented by Carpenter et al.^15^.

**Figure 7:**
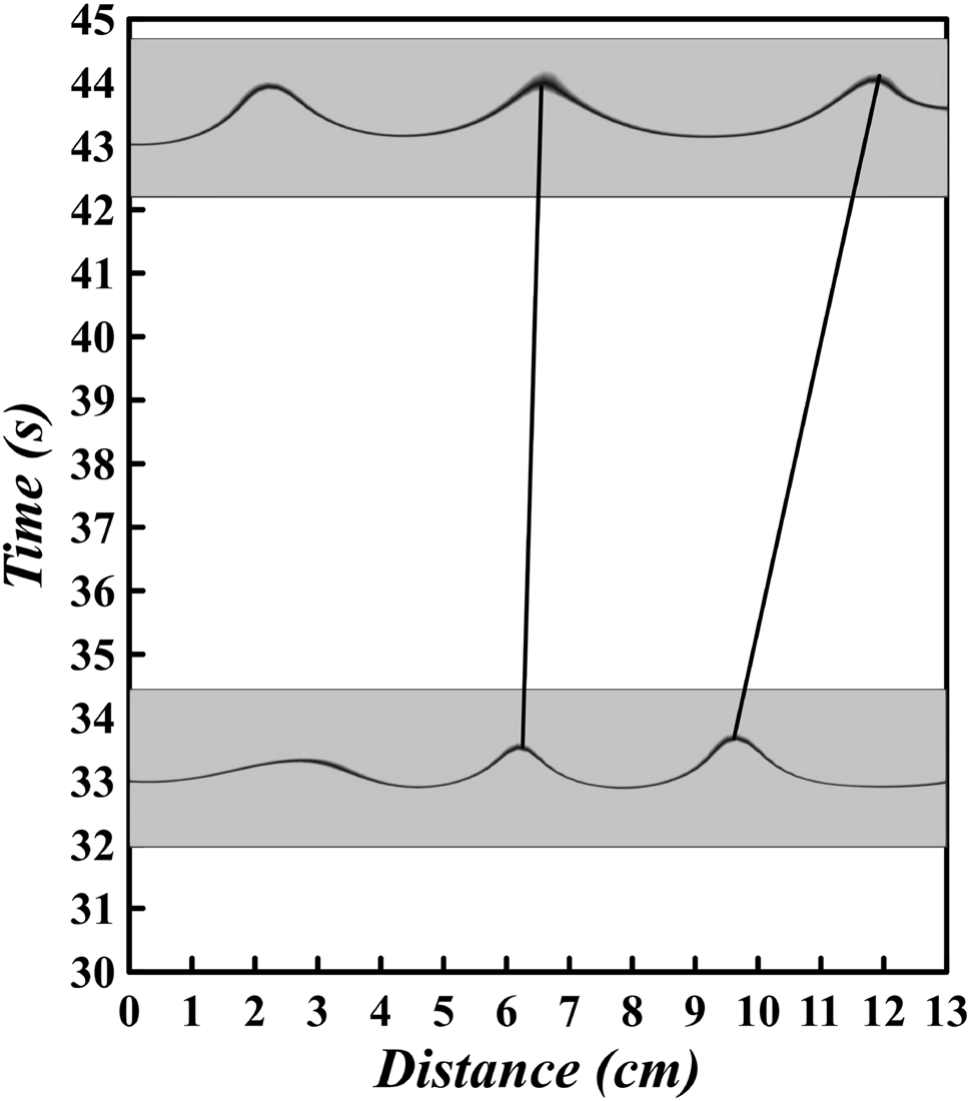
A schematic representation of the Holmboe wave formation and stretching processes, as simulated with the values of $c_{1} = 0.2$, $h^{*} = 0.3$, and $d_{p} = 12$ $\upmu$m, is provided. The interface elevation is demonstrated at two times, keeping pace with the same wave. The horizontal displacement at each time is related to the hindmost crest of the wave. Initially, the wave is represented with a wavelength equivalent to the wavelength of maximum growth, eventually being extended to twice of the original length.

The schematic depicted in Fig. 7 reveals the formation and stretching processes of Holmboe waves with the values of $c_{1} = 0.2$, $h^{*} = 0.3$, and $d_{p} = 12\,\upmu$m. The interface between the IGC and LGC is shown to deviate due to the disparity in speed between the two layers which causes the leading crest to progress further than the rear crest. This divergence in phase speed leads to an elongation in the wavelength along the length of the channel and thus, stretching of Holmboe waves is enabled. Additionally, it can be observed that the amplitude of the Holmboe waves increases qualitatively, as shown in the figure.

**Figure 8:**
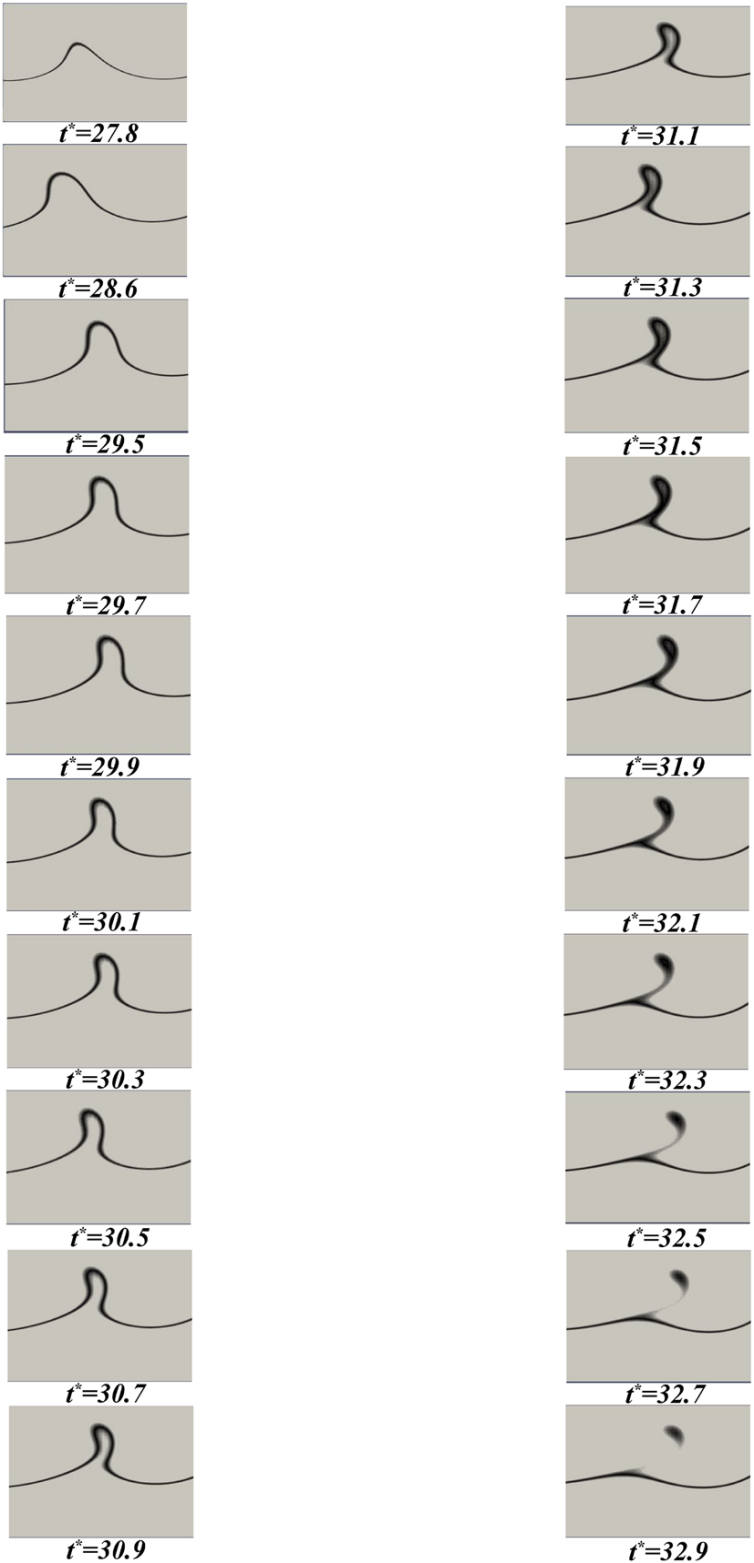
The simulation of the Holmboe wave fluid ejection phenomenon with the respective values of $c_{1} = 0.3$, $h^{*} = 0.3$, and $d_{p} = 12$ $\upmu$m is carried out. The entire procedure is illustrated in a series of sequential images, spanning the time period from $t^{*}=27.8$ to $t^{*}=32.9$.

The asymmetric Holmboe waves are distinguished by the alternating ejection of fluid plumes from the LGC to the IGC. This fluid ejection is the predominant factor responsible for the mixing of the two layers, resulting in a comparable degree of mixing to that of the Kelvin–Helmholtz waves, despite the lower rate of growth of Holmboe waves^43^. An illustration of the wave formation and decay, coupled with the ejection of fluid, for a given set of parameters ($c_{1} = 0.1$, $h^{*} = 0.3$, and $d_{p} = 12\,\upmu$m) is presented in Fig. 8. This figure shows that the wave amplitude reaches its maximum, followed by the ejection of fluid, and finally the wave is decayed. This process of fluid ejection is caused by a pair of vortices with opposite rotational directions. The first of these is attributed to the density difference between the crest of the wave, while the second is the consequence of the shear force acting to roll up the interface between the two layers^44^.

**Figure 9:**
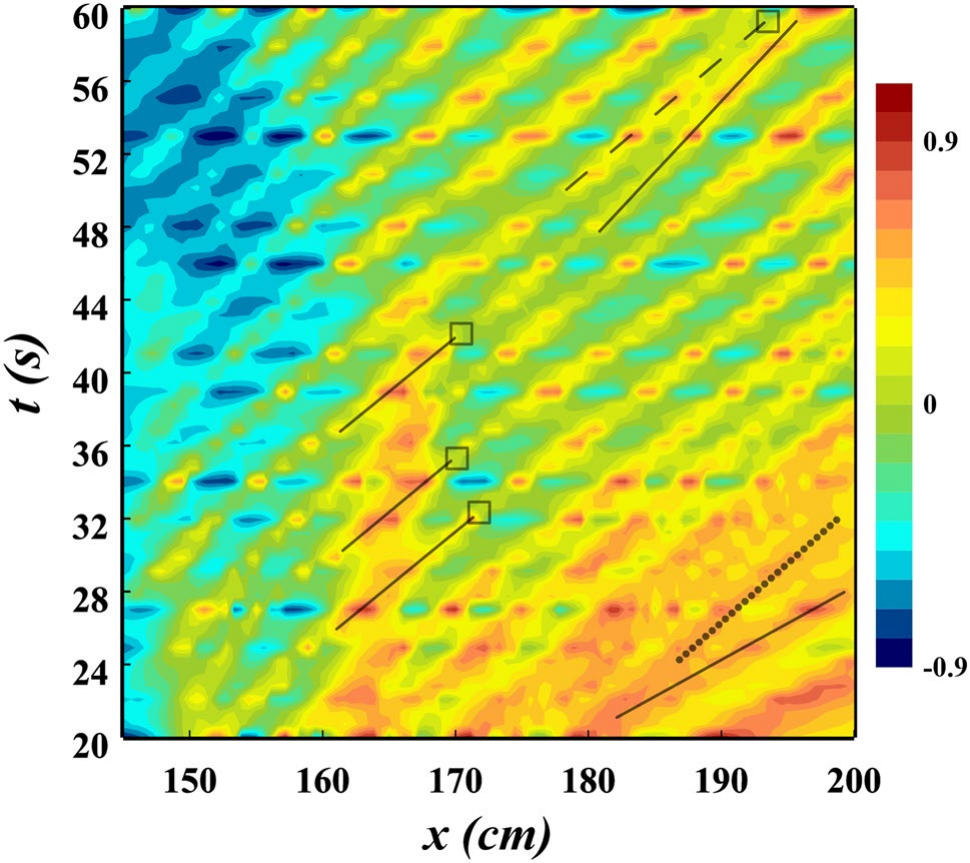
The elevation of the density interface, depicted by shading, is represented by a distinct colour scale, where red indicates a high crest and blue indicates a low trough, for Holmboe waves with $c_{1} = 0.4$, $h^{*} = 0.3$, and $d_{p} = 12$ $\upmu$m.

The $x-t$ characteristic diagram of the density interface elevation in Fig. 9 reveals many of the fundamental features of the wave field. The elevation of the density interface is symbolized by color gradient shading, with higher values (crests) represented by red and lower values (troughs) by blue. The Holmboe instabilities are observed to be present from $x = 150\,$cm to $x = 200\,$cm at $t = 20\,$s, and their amplitude remains relatively constant. As the simulation progresses, the loss of wave crests over time can be observed, which is illustrated by the solid and dashed lines that track the wave crests from $t = 48\,$s to $t = 60\,$s.

The lost wave crests are indicated by the dashed line, while the solid lines are corresponded to crests that persist. On the other hand, It is revealed, from $t = 20\,$s to $t = 32\,$s at the end of the channel (Fig. 9), the wave characteristics display a distinctly different behaviour. In this case, new wave crests are continually being formed as the Holmboe waves travels the channel. Again, this process is highlighted by the tracing of crests using the solid and dots lines. New wave crests that have been formed within the channel are represented by the dots line. These phenomena that were discussed have also occurred in past studies^19,45^, but they were not seen in the IGC.

A decrease in wave amplitude can be observed at multiple points and locations (Fig. 9). These sudden alterations in amplitude are sometimes associated with the release of fluid. In Fig. 9, instances where fluid ejection takes place are recognized by the square markers. Moreover, it is usually seen that the highest waves are more susceptible to the ejection process (Fig. 8), which is further corroborated in Fig. 9. Additionally, it has been found that two or three Holmboe waves can be merged and a considerable amount of LGC fluid is discharged into the IGC, occurring between $x = 170\,$cm and $x = 200\,$cm over a period of 20–36 s (Fig. 9).

### The IGC density

The influence of the density difference between the IGC and the LGC on the characteristics of Holmboe waves, such as wavelength, phase speed, growth rate and wave frequency, is examined by considering two different simulations (PL and PF) in which the IGC density is increased. To quantify the instability, the parameter *R* (the ratio of the shear layer thickness to the thickness of the density layer) and the local Richardson number *J* (defined in Eqs. (16) and (17)) are employed^46^.

16 $$\begin{aligned}&R = \frac{\delta _{v}}{\delta _{\rho }} \end{aligned}$$

17 $$\begin{aligned}&J = \frac{2\Delta \rho g\delta _{v}}{\rho _{l}\Delta U^{2}} \end{aligned}$$

The thicknesses of the shear and density layers are denoted by $\delta _{v}$ and $\delta _{\rho }$ respectively, with $\rho _{l}$ representing the density of the LGC. By employing Eqs. (16) and (17), it is therefore possible to ascertain the boundaries of each instability, thereby constructing a criterion to distinguish between them.

#### R and J

**Figure 10:**
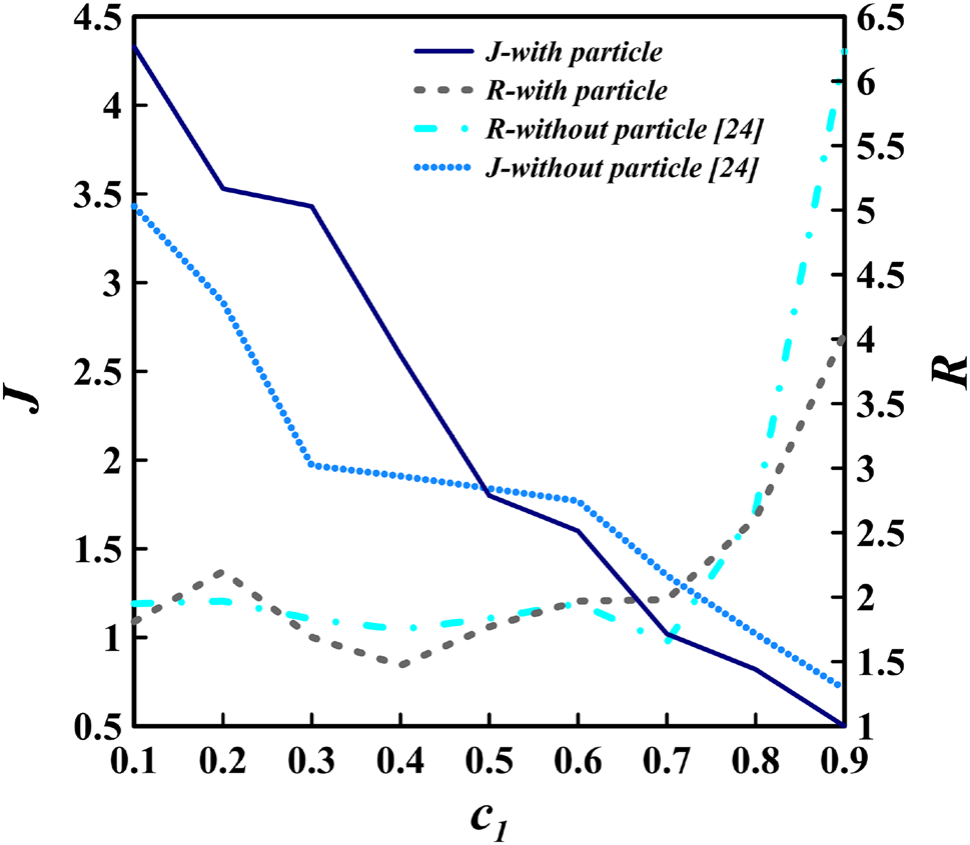
The IGC is studied with two distinct simulations (PL and PF) to determine the *R* (the ratio of the shear layer thickness to the thickness of the density layer) and *J* (Richardson number) parameters in a variety of densities ($c_{1}$), with a fixed value of $h^{*} = 0.3$ and $d_{p} = 12\mu m$ at $t^{*} = 49$^24^.

**Figure 11:**
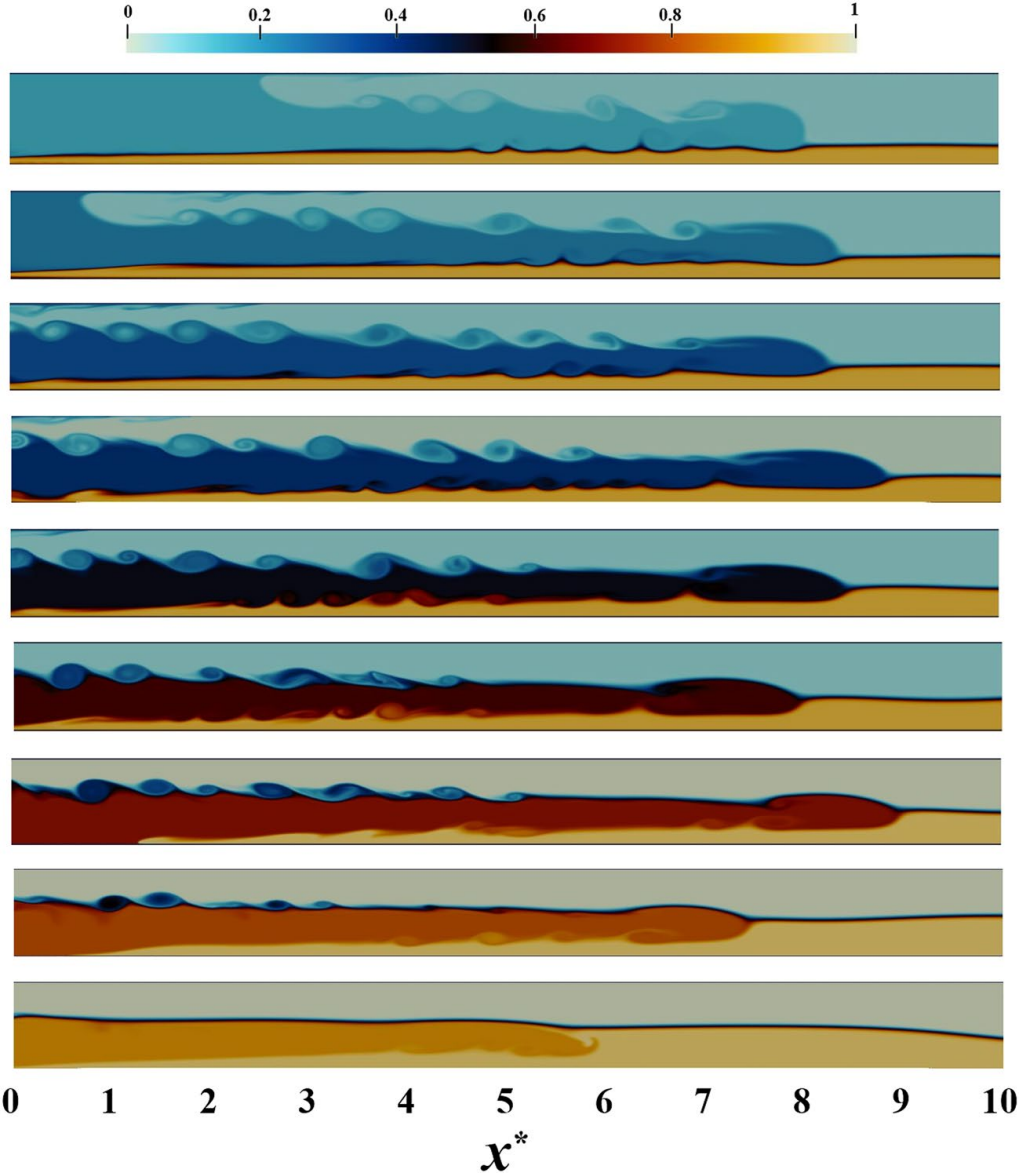
The IGC movement trends for a variety of densities are investigated assuming a particle diameter of 12 $\upmu$m at $t^{*} = 27$ and $h^{*} = 0.3$ (with the IGC densities varying from 0.1 to 0.9 from top to bottom). The alteration in the relative density of IGC, UGC and LGC, as represented by the color band, is evidenced in the range of colors observed. This density field has a range of values from 0 to 1. It is evident that Holmboe waves exist across a wide range of densities.

By computing the mean values of *R* and *J*, Fig. 10 is depicted for two distinct simulations (PL and PF). As evidenced, with the increase of the IGC density (decrease in the density difference), the value of the *J* diminishes in both cases and the *R* parameter increases, which is consistent with the findings of Khavasi and Firoozabadi^26,47,48^. According to the linear stability theory and furthermore laboratory experiments, it is postulated that *J* augments as the density difference grows^26,47,48^. The occurrence of Holmboe waves is resultant from this trend. As expected, Holmboe waves become less likely to exist in high densities ($c_{1}>0.8$) as *J* decreases; however, since the value of *R* is greater than its critical value and *J* is greater than 0.25, the waves are still observed in this range of $c_{1}$. Figure 11 portrays the variation of the IGC for different densities with the assumption of PL. It is discernible that the Holmboe waves persist in all cases, even at densities greater than 0.8, at the boundary between the IGC and the LGC. This relationship between a decline in the probability of Holmboe waves and an increase in the IGC density can be explained by the fact that Holmboe waves are generated under conditions where *J* is high (the buoyancy force being dominant in such conditions)^49^, and therefore, an increase in the IGC density leads to an increase in the inertial force and a decrease in the buoyancy force.

An analysis of Fig. 10 with regards to the introduction of particles to the IGC reveals that for $c_{1}<0.5$, the values of *J* for the PL IGC are larger than their counterparts in the PF IGC; however, when $c_{1}>0.5$, this trend is reversed. This phenomenon in the diagram can be ascribed to the fact that the speed of the IGC (due to the presence of the particles) and the LGC (as a result of sedimentation) is reduced when particles are added to the IGC, thereby leading to a higher *J* value for the PL at a constant density. The interesting results of the density difference constant reduction for $c_{1}>0.5$ shall be explored in future research. It can be deduced from the general trend that the difference of Richardson number values for $c_{1}<0.5$ between two simulations (PL and PF) is decreasing, and no specific trend is observed in the *R* parameter with the addition of particles.

#### The frequency

**Figure 12:**
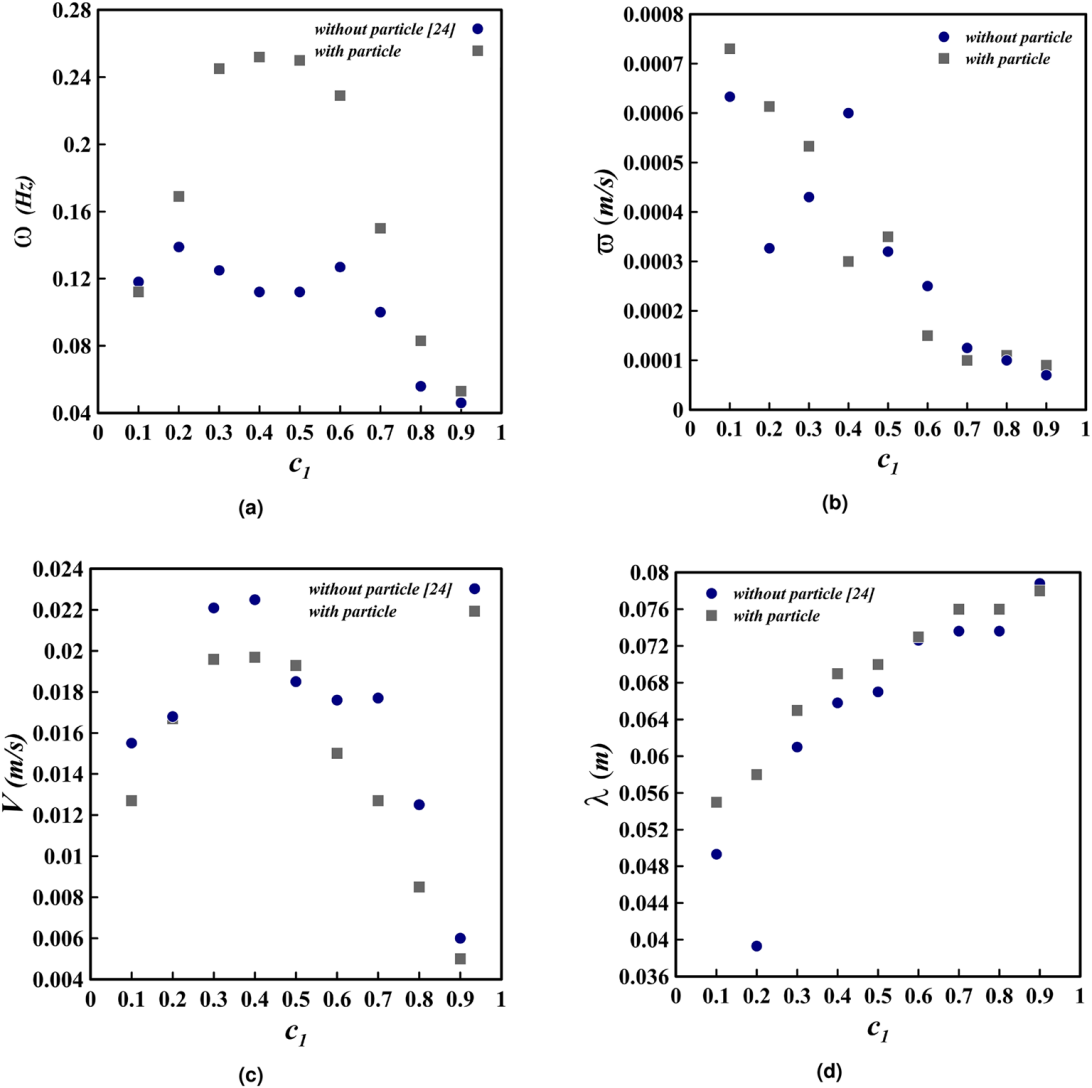
Holmboe waves average frequency (**a**), growth rate (**b**), phase speed (**c**) and wavelength (**d**) in different density range of the IGC for two distinct simulations (PL and PF) with values of $h^{*} = 0.3$, and $d_{p} = 12\mu m$ are indicated^24^.

Figure 12a illustrates the average frequency of Holmboe waves over a period of 38 s in different densities of IGC. An increase in IGC density for the PF simulation is observed to be associated with a two-part alteration in wave frequency, with the highest increment in frequency occurring when $c_{1}=0.2$, as is clearly demonstrated in the figure. Meanwhile, in the PL simulation, the process is experienced only once, with the highest frequency of 0.252 Hz occurring at $c_{1}=0.4$.

By altering the IGC density from $c_{1}=0.1$ to $c_{1}=0.9$, the frequency of waves fluctuates from 0.118 to 0.046 Hz in the PF simulation and from 0.112 to 0.053 Hz in the PL simulation. Moreover, as evidenced in Fig. 12a, the frequency of Holmboe waves along the channel is augmented, as can also be discerned in Fig. 11. To put it another way, the number of Holmboe waves increase along the channel from left to right in various locations.

Examining Fig. 11 in the context of PL simulation reveals the obvious trend in Fig. 12a. That is to say, the number of Holmboe waves increases with an increase in density, before eventually diminishing. It is further evident that the simulation with $c_{1}=0.1$ exhibits a higher number of Holmboe waves than the simulation with $c_{1}=0.9$.

**Figure 13:**
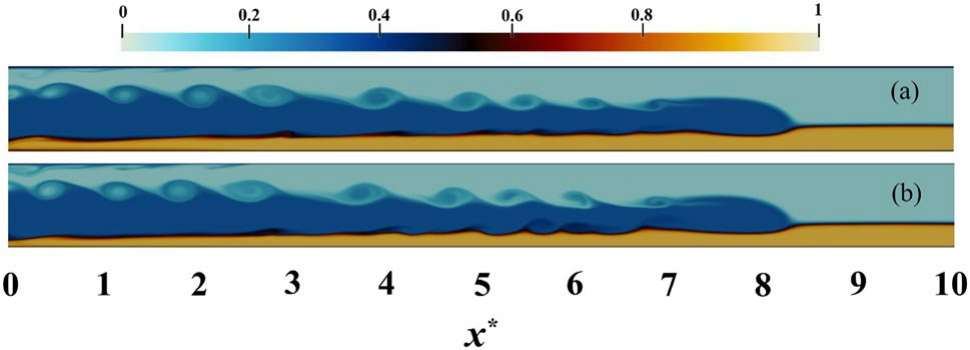
The IGC movement trends for $c_{1}=0.3$ with the value of $h^{*} = 0.3$ at $t^{*} = 27$. (**a**) simulation with PF assumption, (**b**) simulation with PL assumption. The variations in color in the density field, ranging from 0 to 1, signify a change in the relative densities of the IGC, UGC and LGC, as represented by the color band.

Upon comparison of two PL and PF simulations, it can be observed that the frequency of waves in the PL case is higher than the PF case in all simulations, with the exception of $c_{1}=0.1$. Nevertheless, these discrepancies are of little significance in 4 different densities ($c_{1}=0.3,\, 0.4,\, 0.5,\, 0.6$). This statement can also be seen in Fig. 13, which illustrates $c_{1}=0.3$ in two PL and PF simulations. The rise in the PL case frequency is substantial, as the inertia of the IGC is decreased with the introduction of particles and, in accordance with Eq. (17), the likelihood of the occurrence of Holmboe waves is augmented.

#### The growth rate

Figure 12b is the result of computing the average growth rate of Holmboe waves for PL and PF simulations at varying densities of the IGC. As depicted in the figure, the Holmboe waves growth rate varies from 0.00009 to $0.00073\,$m/s under the PL assumption, and from 0.00007 to $0.00063\,$m/s under the PF assumption, as the difference in density between the IGC and the LGC decreases.

The results indicated by Fig. 12b demonstrate that the growth rate of Holmboe waves in the IGC is not consistent with the local Richardson number; this is in accordance with the linear stability theory of Khavasi and Firoozabadi^47,48^. The general trend of the figure shows that, with an increase in the Richardson number for both PL and PF cases, the growth rate initially increases, then diminishes and rises again. This pattern of fluctuation can be interpreted as that when the Richardson number grows, the buoyancy force increases, although not to a degree sufficient to surpass the Holmboe waves growth rate. Nevertheless, as the buoyancy force continues to augment, it overpowers the inertial force and results in the reduction of the waves growth rate.

By analyzing the results of both PL and PF simulations illustrated in Fig. 12b, it is apparent that the addition of particles with a diameter of $12\, \upmu$m does not significantly impact the growth rate. This is evidenced by the higher values of the growth rate in the PF case for $c_{1}=0.4,\, 0.6,\, 0.7$, while the values of the PL case are higher in the other instances. Furthermore, comparison of the two PL and PF assumptions at $c_{1}=0.3$ in Fig. 13 reveals that the Holmboe waves growth rate is greater in the PL assumption. Additionally, examination of the growth rate values in Fig. 12b for $c_{1}=0.3$ demonstrates that the Holmboe waves growth rate in the PL assumption is $23.95\%$ higher.

#### The phase speed

The average phase speed of Holmboe waves depicted in Fig. 12c for both PL and PF simulations in various IGC densities reveals that the waves attain their highest speed at $c_{1}=0.4$ ($0.0225\,$m/s in the PF case and $0.0197\,$m/s in the PL case) and the lowest speed at $c_{1}=0.9$ ($0.006\,$m/s in the PF case and $0.005\,$m/s in the PL case). Furthermore, the reduction of the density to $c_{1}=0.4$ leads to an increment of the phase speed by $275\%$ and $294\%$ for the PL and PF assumptions, respectively, thereby indicating the influence of the increasing density difference on the phase speed. The qualitative analysis of the data in Fig. 12c shows that the phase speed initially augments and then reduces with the decrease of IGC density, without exhibiting a discernible pattern. This outcome is in agreement with the experiments of Khavasi and Firoozabadi and Zhou and Lawrence, while the results of linear stability theory indicate that the Holmboe wave phase speed is always proportional to the Richardson number^13,26,47,48^. The addition of particles to the IGC reduces its speed, thereby decreasing the distance it travels, which in turn results in a decrease in the speed of the LGC due to the deposition of particles. This is attributed to the reduced inertia of the current, leading to a decrease in the phase speed of Holmboe waves. The exception being the $c_{1}=0.5$ assumption, which yields the opposite result. By analyzing Fig. 12c, it can be determined that the mean and maximum rate of fluctuation in relation to the consequence of particles presence on the wave phase speed are 19.7 and 47

#### The wavelength

By assessing the interface between the IGC and the LGC in two separate simulations (PL and PF) for varying IGC densities, Fig. 12d depicts the average wavelength of Holmboe waves. The mean value of these simulations was calculated as 6.7 cm, which is lower than the value of 10 cm determined by Tedford et al. through examining symmetric Holmboe waves^12^.

The Richardson number decreases as the difference in density between two layers diminishes, which is evidenced in Fig. 12d unless $c_{1}=0.2$. This reduction, in turn, leads to an increase in the wavelength of Holmboe waves. The disparity between the speeds of the two layers results in a difference in the phase speed of the two crests of the Holmboe wave. As the inter-layer gradient of density intensifies, the speed difference between the two layers amplifies, thus creating a greater difference in the phase speed of the two crests, and consequently, a larger wavelength. This conclusion has been corroborated in both laboratory experiments by Khavasi and Firoozabadi and Zhou and Lawrence^13,26^.

The results contained in Fig. 12d demonstrate that, with the exception of $c_{1}=0.9$, the addition of particles to the IGC causes the wavelength to enhance. Further, the average change rate of the wavelength for both PL and PF assumptions is $4.325\%$, and it can be seen that the incorporation of particles with a diameter of $12\,\upmu$m does not have a considerable impact on the increase of the wavelength (apart from $c_{1}=0.2$).

### Particle size effect

The influence of particle size increase on the Holmboe instability is studied in this section, considering the dimensionless settling velocity of particles ($V_{f}$) calculated according to the Stokes equation (Eq. 12). It is assumed that the material of the particles is Kaolin and the initial hypothesis diameter size is $20\, \upmu$m, which corresponds to a settling velocity of $V_{f}=0.00294$. The variation in settling velocity is proportional to the change in particle size, with larger particles having a higher settling velocity value. The $V_{f}$ values for the particles utilized in the present work are provided in Table 3, considering a IGC density of $c_{1}=0.3$ and a dimensionless height of the LGC of 0.3.

**Table 3:** Dimensionless settling velocity values ($V_{f}$) for particles used in this study.

$d_{p}$	$12\, \upmu$m	$20\, \upmu$m	$30\, \upmu$m
$V_{f}$	0.00106	0.00294	0.00661

#### R and J

**Figure 14:**
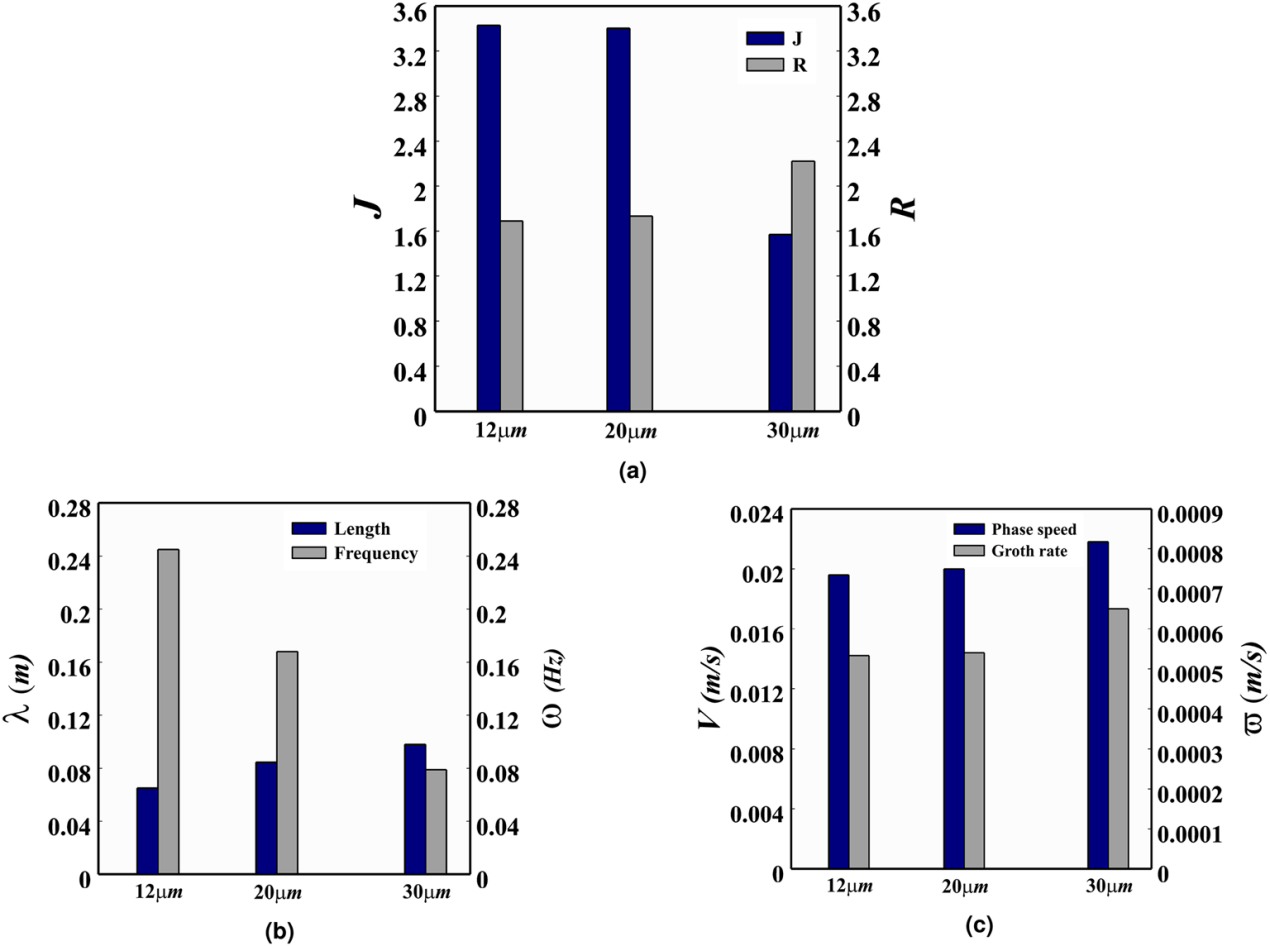
The mean values of *R* and *J*, frequency and wavelength, and phase speed and growth rate for the PL IGC, with a density of $c_{1}=0.3$ and $h^*=0.3$, are displayed in (**a**–**c**), respectively. Particles with diameters of $12\, \upmu$m, $20\, \upmu$m, and $30\, \upmu$m are taken into consideration.

The Fig. 14a results are obtained by calculating the mean values of *R* and *J* at $t^{*}=49$ for $12\, \upmu$m and $20\, \upmu$m diameter, and at $t^{*}=12$ for $30\, \upmu$m diameter. As alluded to in the section on the effect of IGC density, an increased number of particles in the IGC causes its speed to reduce, while the distance it traverses diminishes. Furthermore, the LGC speed is subject to a gradual decrease due to the deposition of particles. The settling velocity of particles is higher for $30\,\upmu$m diameter (see Table 3), thus leading to a more pronounced reduction in the speed of both the IGC and the LGC when compared to the other two diameters. After $t^{*}=40$, the LGC is no longer visible at the bottom of the channel due to its settling. Thus, the values of *R* and *J* were measured at $t^{*}=12$ for the $30\, \upmu$m simulation. Analysis of Table 3 reveals that the settling velocity of particles has a marked increase of 177.36% when the particle diameter is increased from 12 to 20 $\upmu$m, and an even more substantial increase of 523.58% when the particle diameter is increased from 12 to 30 $\upmu$m.

**Figure 15:**
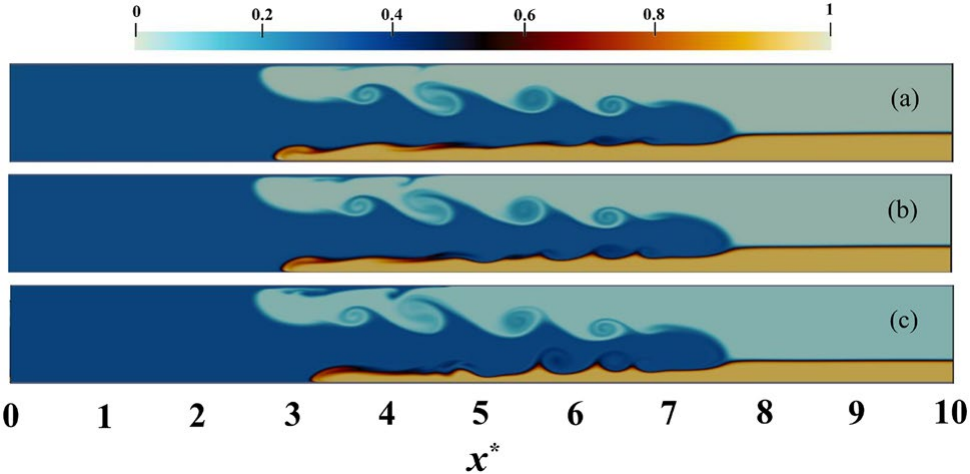
An examination of the IGC motion trends for a constant value of $c_{1} = 0.3$ under the PL assumption at $t^{*} = 12$ and $h^{*} = 0.3$ with respect to the three micro-meters $12\, \upmu$m (**a**), $20\, \upmu$m (**b**) and $30\, \upmu$m (**c**), reveals a distinct variation in color, which is suggestive of a modification in the comparative denseness of IGC, UGC and LGC, as represented by the color band. The density field in this case is observed to vary between 0 and 1.

As indicated in Fig. 14a, a notable inverse correlation is present between the values of *R* and *J* and the particle diameter. A notable point is that a particle diameter increase above $20\,\upmu$m has a significant effect on *R* and *J* values. It is discernible that the values of *R* and *J* remain relatively constant as the particle diameter ranges from $12\,$ to $20\,\upmu$m, however, when the diameter expands to $30\, \upmu$m a substantial augmentation is observed, with *R* reaching 2.22 and *J* reaching 1.57. Furthermore, it is noteworthy that the Richardson number reduction caused by the rise in particle diameter reduces the likelihood of the emergence of Holmboe waves which can be observed in Fig. 15.

#### Frequency and wavelength

By analyzing Fig. 14b, it is evident that the Holmboe waves frequency declines with the increase in the particle diameter. This is largely attributable to the corresponding reduction in the Richardson number, which reduces the likelihood of the waves presence. Consequently, the frequency decrease is substantial; the waves frequency drops by $67.75\%$ when the particle diameter goes up from 12 to $30\, \upmu$m. This finding corroborates the results of Tedford et al., who conducted a frequency survey along the channel for a specific simulation ($c_{1}=0.3$)^12^.

Besides the average frequency, Fig. 14b elucidates the average wavelength of Holmboe waves in the simulations carried out in this section, which is found to be 8.4 cm, a value that is more proximate to the one reported by Tedford et al. (10 cm)^12^. As demonstrated in the graph, a decrease in the Richardson number leads to an amplification in the wavelength of Holmboe waves.

The Holmboe wave’s average wavelength in the PF simulation with a coefficient of $c_{1}=0.3$ is 6.1 cm. This can be contrasted with the PL case in Fig. 14b, where the wavelength for a particle diameter of $30\, \upmu$m is 9.8 cm and for a diameter of $12\, \upmu$m is 6.5 cm. This significant disparity between the two cases can be further analysed in terms of frequency. Therefore, it can be ascertained that small particles do not have a substantial impact on the Holmboe instability. Additionally, an increase in particle diameter leads to an acceleration in sedimentation, resulting in a decrease in the speed of the LGC. Nevertheless, the speed of the IGC remains relatively unchanged (as seen in Fig. 15).

#### Phase speed and growth rate

Figure 14c illustrates the average Holmboe wave phase speed for the simulations detailed in Table 3. It can be perceived that, as the Richardson number declines (and thus the $V_{f}$ increases), the phase speed reduces. Furthermore, the influence of particles with diameters larger than $20\,\upmu$m is also evident. Specifically, an increase in the particle diameter size from 12 to $20\,\upmu$m leads to a rise in the phase speed of $0.0004\,$m/s, while a further increase in the particle diameter size from 12 to $30\,\upmu$m results in a phase speed increment of $0.0022\,$ m/s.

Examining Fig. 14c, an upward trend in the growth rate of Holmboe waves is observed as the $V_{f}$ increases. This denotes that only small-sized particles have little to no effect on the instability of the current; however, with a further rise in the $V_{f}$ (particles with a diameter of $30\,\upmu$m), the growth rate is significantly higher than that of the PL simulations with diameters of $12\,\upmu$m and $20\,\upmu$m. Notably, when the diameter is increased up to $20\, \mu m$, the growth rate only increases by $1.3\%$, but when the diameter is increased up to $30\,\upmu$m, this value rises by $21.95\%$.

#### Q criterion

The Q criterion is utilized to demonstrate the characteristics of vortical structures within the channel, thereby making it possible to identify locations where fluid rotation is predominant and thus to determine the vorticity strength. Evidently, the balance between the strain rate and the vorticity power is evidenced by this criterion, with its positive values indicating regions where the flow rotation power is more forceful than the strain rate (deformation). The magnitude of the scalar Q is established in accordance with the second invariant of the velocity gradient tensor (Eq. 18)^28^.

18 $$\begin{aligned} Q = \frac{1}{2}(\Omega _{ij}\Omega _{ji} - S_{ij}S_{ji}) \end{aligned}$$

The symmetric and asymmetric components of $\nabla u$, denoted as $S_{ij}$ and $\Omega _{ij}$ respectively, can be expressed as follows^28^:

19 $$\begin{aligned} S_{ij}= & {} \dfrac{\partial u_{ij}}{\partial x_{ij}}+\dfrac{\partial u_{ij}}{\partial x_{ij}} \end{aligned}$$

20 $$\begin{aligned} \Omega _{ij}= & {} \dfrac{\partial u_{ij}}{\partial x_{ij}}-\dfrac{\partial u_{ij}}{\partial x_{ij}} \end{aligned}$$

**Figure 16:**
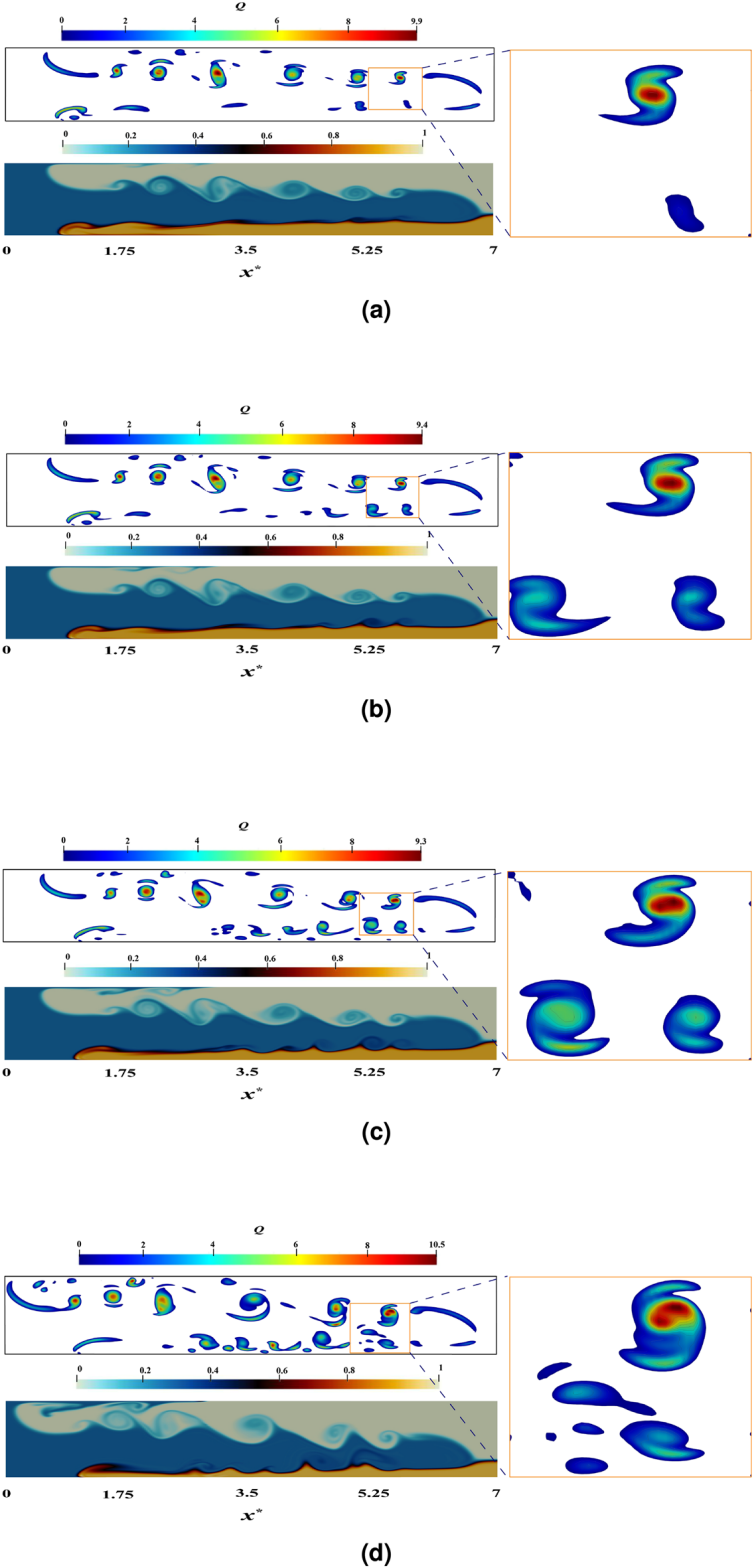
The Q-criterion contours of simulations PL and PF ($12\, \upmu$m, $20\, \upmu$m and $30\, \upmu$m) with $c_{1}=0.3$ and $h^{*} = 0.3$ at a dimensionless time of 15 are depicted in terms of a color variation in the density field, ranging from 0 to 1, which is indicative of a shift in the relative densities of the IGC, UGC and LGC as evidenced by the color band.

Figure 16 exhibits the Q contours for multiple simulations conducted in four distinct states (namely, PF, $12\, \upmu$m, $20\, \upmu$m, and $30\, \upmu$m for $c_{1}=0.3$ and $h^{*} = 0.3$) at a dimensionless time of 15. It can be observed that, within the zoom zones, the diameter of the Q contours diminishes as its value grows, a phenomenon known as the diffusion of vorticity. Stronger eddies, possessing a smaller diameter, propagate their rotational character symmetrically and induce the emergence of weaker eddies with a larger diameter forming a shell-like structure around them. Consequently, the vorticity diffusion leads to the generation of concentrically rotating tubes, whose rotational power decreases as it moves further away from its epicenter.

Analyzing the interface between the IGC and LGC, the site of Holmboe wave formation, it can be displayed that, in the lack of particles (Fig. 16a), the quantity of eddies with a rotational power (the number of Q contours at the interface in the $30\, \upmu$m state: Fig. 16d) diminishes to the extent that some of them change into irregular forms and eventually dissipate. Additionally, as seen in the inset zoom zones, the strength of the remaining eddies also decreases. In essence, due to the presence of particles, the amount of vorticity at the interface between the two currents declines.

The growth in diameter of particles in IGC leads to a decrease in speed, albeit at a rate less than that observed in LGC (see Fig. 15); the latter therein disappearing after 40 s. This disparity results in a velocity gradient and a subsequent elevation of vorticity accompanying the enlargement of the particles’ diameter. The alteration of velocity gradients due to the introduction of particles results in the modification of the magnitude of vorticity and the shape of vortical structures at the interface, implying a logical consequence of a greater intensity of vortices.

A more thorough examination of Q can reveal additional noteworthy information. Through an analysis of the velocity vectors in the “wave evolution” section, it was revealed that a region of high vorticity was formed in the lee of the waves crest, with the dimension of this region being larger for Kelvin–Helmholtz waves than that of Holmboe (see Fig. 6a). This statement is further supported by the investigation of the Q contours, which indicate that the vorticity produced by Kelvin–Helmholtz waves is greater, resulting in a larger region being formed.

Ultimately, it is particularly noteworthy that particles with a diameter exceeding $30\, \upmu$m have a more substantial impact on the evaluation of the Q criterion for Holmboe waves than other states, as the amount of vorticity at the origin of these waves undergoes considerable variations in this condition.

### The inclined bed

Research into the instability of stratified currents has largely assumed the current to be moving on a flat bed; however, in the natural environment, some currents, such as those entering reservoirs of dams, flow over an inclined bed. The gradient of the bed may influence the nature of interface instabilities. Thus, this study aims to analyze the behavior of the interface between the IGC and the LGC when the slope of the channel bed is changed to $2^{\circ }$, $4^{\circ }$, and $6^{\circ }$, with a constant value of $c_{1}=0.3$, $h^{*}=0.3$, and in the absence of particles.

**Figure 17:**
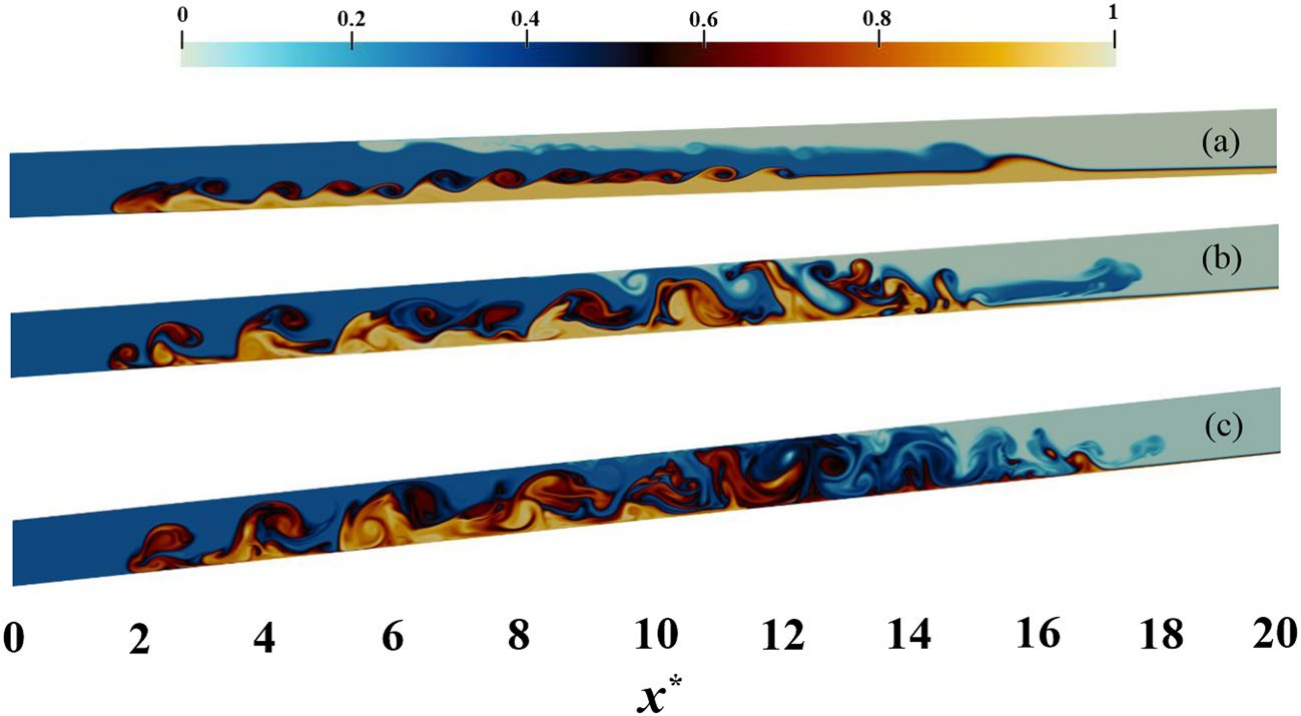
The IGC movement trends for $c_{1}=0.3$ with the PF assumption at $t^{*} = 27$ and $h^{*} = 0.3$ are visually represented by three distinct slopes that represent (**a**) $2^{\circ }$, (**b**) $4^{\circ }$, and (**c**) $6^{\circ }$ respectively. The variation in color is an indication of a modification of the relative density of IGC, UGC and LGC, which varies between 0 and 1.

The linear stability theory elucidates that as the slope steepens, the range of instability increases and the Miles–Howard criterion is no longer applicable for $J > 0.25$^47,48^. Table 4 indicates a decrease in the Richardson number as the slope rises, signifying the current instability and an expanded instability range, which is also perceptible in Fig. 17. This outcome is in agreement with the findings of Negretti^46^. To justify this issue, it is well established that buoyancy and inertia are the fundamental forces governing the double-layer current. An increase in the bed slope results in an augmentation of the inertial force which disrupts the stability of the current, whilst simultaneously decreasing the stabilizing buoyancy force. Moreover, results from the study suggest that the growth of Kelvin–Helmholtz waves enhances with the elevation of the bed slope. In contrast, for Holmboe waves, the slope effect is considerably disparate, due to their emergence at high Richardson numbers. Consequently, Holmboe waves are not observed in the geometry of the current study (Fig. 17).

**Table 4:** Average values of *J* for the PF IGC on inclined bed.

Slope	0 Deg	2 Deg	4 Deg	6 Deg
J	1.97	0.85	0.3	0.28

### The transition range in the IGC

**Figure 18:**
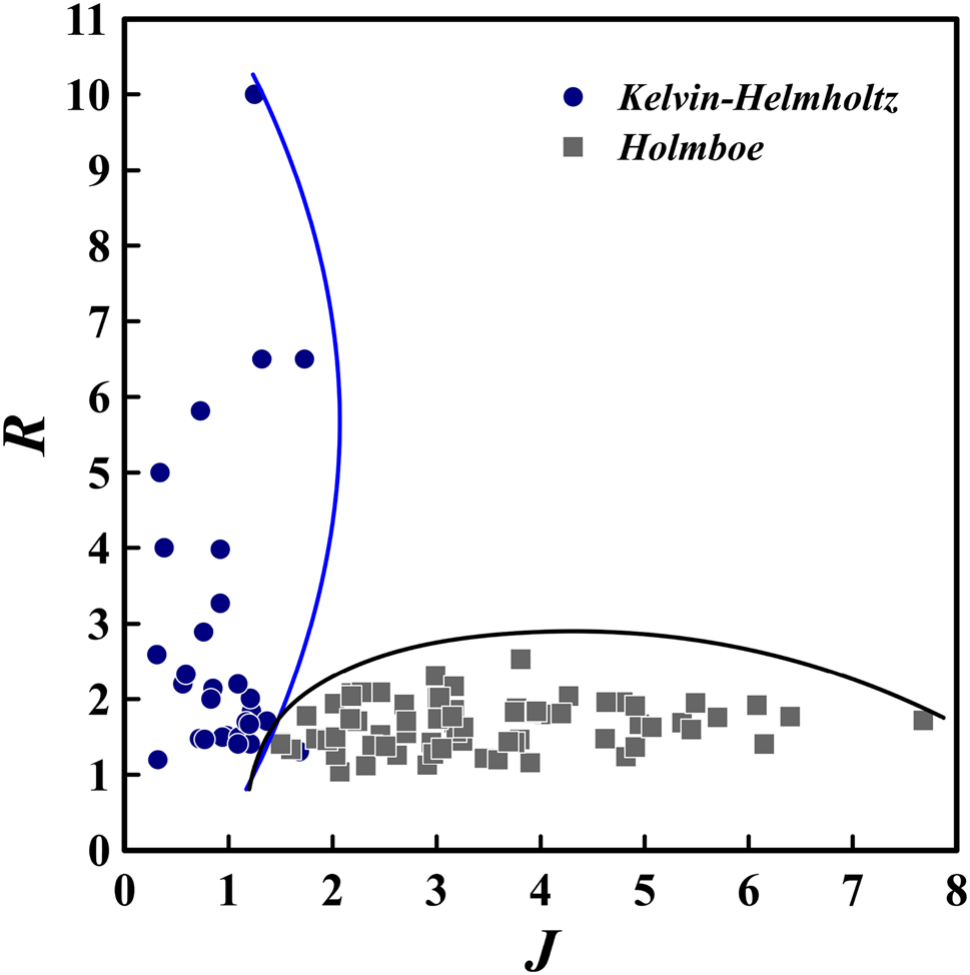
The *J*–*R* diagram for the IGC (PL and PF cases) reveals the presence of Holmboe instabilities in the range of $1.6< J < 7.68$ and $1< R < 2.4$, and Kelvin–Helmholtz instabilities within the range of $0.32< J < 1.35$ and $1.5< R < 10$. The delineation between Holmboe and Kelvin–Helmholtz waves is depicted by the curved lines.

It can be deduced from the aforementioned statements that instability type is contingent upon the two parameters of *R* and Richardson number. Consequently, a range may be ascertained to identify the instability and a criterion to differentiate between instabilities can be procured. By calculating the local values of *R* and *J* for all relevant simulations at each station where Holmboe and Kelvin–Helmholtz waves arise, a range of transition from Kelvin–Helmholtz to Holmboe instability for the IGC in the current study can be introduced (see Fig. 18).

Examining Fig. 18, Holmboe waves are observed to be more likely to occur in higher Richardson numbers and smaller *R* values than Kelvin–Helmholtz waves. Specifically, the most frequent Holmboe waves appear at roughly $J > 1.6$ and $R > 1$. Khavasi and Firoozabadi identified asymmetric Holmboe waves in their experiments when $R > 1$, though their calculations indicate that Holmboe waves require $J > 10$^26^. At $J < 1.5$ and $R > 1.5$, Kelvin–Helmholtz waves are seen (with the exception of two cases). It is evident from Fig. 18 that Holmboe waves are more prevalent at high Richardson numbers, whereas Kelvin–Helmholtz waves are more common at lower Richardson numbers.

Despite the calculation technique (numerical, experimental, or linear stability theory) employed, other factors may influence the instabilities of stratified currents. These include the inter-particle interactions, particle-fluid interactions, the effects of sidewalls, the disparity in density, the gradient of the bed, and the restricted height of the ambient fluid above the current. Consequently, these factors, along with the specific conditions of the simulation, can account for the discrepancy in the transition criteria from Kelvin–Helmholtz to Holmboe waves.

Looking again in Fig. 18 shows that at the same *J* and *R*, both of Kelvin–Helmholtz and Holmboe waves can be occurred. This phenomenon is happened because of the uncontrolled disturbances in the simulations. It means, the average current conditions may be similar in two cases, but the noises and disturbances caused by other factors are not the same (have different amplitudes and wavelengths). For theoretical investigations, it has been perceived that with the same *J* and *R*, the current may be stable in one wavelength but unstable in another wavelength^47,48^. Therefore, the fluctuations presence with different wavelengths can be the factor of the current stability or instability in conditions where the current average characteristics are alike.

Finally, it can be determined from Fig. 18 that a demarcation exists between Holmboe and Kelvin–Helmholtz instability. Holmboe instability is perceptible, with only two exceptions, in the region of $1.6< J < 7.68$ and $1< R < 2.4$, and Kelvin–Helmholtz instability is discernible, apart from two exceptions, in the span of $0.32< J < 1.35$ and $1.5< R < 10$.

## Conclusion

The investigation of Holmboe waves in intrusive gravity currents (IGCs) was conducted with the assistance of the LES for two PL and PF assumptions, the outcomes of which were subsequently discussed.

Initially, an examination of the structure and properties of Holmboe waves was conducted under the PL scenario in order to determine the methods of formation and the characteristics of these waves in IGCs. It was determined that the region of influence of the Holmboe waves was smaller than that of Kelvin–Helmholtz waves, based on an examination of the velocity vectors. Additionally, closed streamlines were observed in the high vorticity region where the Kelvin–Helmholtz wave was generated. Secondary rotations were also created in the Holmboe waves. Furthermore, the evaluation of the interface deviation between the IGC and the LGC revealed that, as the two crests of Holmboe waves travelled along the channel, they were separated due to the difference in phase speed, leading to an increased wavelength over time. The phenomenon of fluid ejection in Holmboe waves was also studied, wherein the most prominent feature was observed to be a sudden decrease in the waves amplitude, which was visible in the *x*–*t* diagram. This decrease followed an enhancement in the waves amplitude until the moment of fluid ejection, when the waves had their highest amplitude. Interestingly, the wave crest was seen to occasionally disappear over time, sometimes to be replaced with a new crest.

In the following, the study looked at how the IGC density variation impacted the characteristics of Holmboe waves. It was observed that as the IGC density increased, the Richardson number decreased and the *R* parameter increased. Additionally, the frequency of the waves first rose and then dropped. Wavelength also increased as the IGC density enhanced, with an average wavelength of around 6.7 cm in the simulations. The growth rate and phase speed, however, did not show a clear trend with the changes in density. The conclusion was that the difference in density between the IGC and LGC had an effect on the Holmboe waves characteristics.

The presence of particles and an augmentation of their diameter had a considerable effect on this study, resulting in noteworthy outcomes. Results were obtained by analyzing the effects of particle size difference on Holmboe wave properties in the IGC. The *R* and *J* values had an opposite reaction to larger particle sizes and the wave frequency decreased. On average, the wavelength was 8.4 cm. The settling velocity of the IGC was larger, causing an increase in the wavelength. The growth rate and phase speed of the Holmboe waves also augmented with the increase of particle size. The key finding was that particles of 20 microns or less had a minimal effect on Holmboe instability. Moreover, noteworthy findings were obtained when analyzing the Q contours in the context of particle existence. Adding particles to the IGC and augmenting their size generated a velocity gradient between the IGC and LGC, which was responsible for an increase in the amount of vorticity in the interface. It was also observed that Kelvin–Helmholtz waves possess a greater degree of vorticity than Holmboe waves.

The bed slope angle being increased up to $6^{\circ }$ yielded major outcomes. This raised the Richardson number, making the current more unstable. The findings showed that as the angle of the bed increased, the growth of Kelvin–Helmholtz waves increased, while Holmboe waves were not present at all in the interface between the IGC and LGC.

To sum up, by analyzing the typical conditions in the sites where Holmboe and Kelvin–Helmholtz waves appear, the *J* and *R* values have been calculated for all of the simulations. The outcome of this analysis has enabled us to mark out a boundary for Holmboe and Kelvin–Helmholtz instabilities in the *J*–*R* diagram for the IGC. Holmboe instabilities were found in the range of $1.6< J < 7.68$ and $1< R < 2.4$, while Kelvin–Helmholtz instabilities were identified in the range of $0.32< J < 1.35$ and $1.5< R < 10$.

## Data Availability

The computing core of the project (i.e., the solver) is developed by (S.R.D.) and is online available at https://github.com/SadeghRostami1996/denseInterFoamBiv.git. Further information on the applied boundary and initial conditions, and the geometry are available upon request from (E.K.).

## Abbreviations

LES: Large Eddy Simulation
DNS: Direct Numerical Simulation
RANS: Reynolds Averaged Navier-Stokes
WALE: Wall-Adapting Local Eddy-Viscosity
PF: Particle-Free
IGC: Intrusive Gravity Current
LGC: Lower Gravity Current
UGC: Upper Gravity Current
PSD: Power Spectra Diagram
PL: Particle-Laden
